# A General Pairwise Interaction Model Provides an Accurate Description of *In Vivo* Transcription Factor Binding Sites

**DOI:** 10.1371/journal.pone.0099015

**Published:** 2014-06-13

**Authors:** Marc Santolini, Thierry Mora, Vincent Hakim

**Affiliations:** Laboratoire de Physique Statistique, CNRS, Université P. et M. Curie, Université D. Diderot, École Normale Supérieure, Paris, France; Macquarie University, Australia

## Abstract

The identification of transcription factor binding sites (TFBSs) on genomic DNA is of crucial importance for understanding and predicting regulatory elements in gene networks. TFBS motifs are commonly described by Position Weight Matrices (PWMs), in which each DNA base pair contributes independently to the transcription factor (TF) binding. However, this description ignores correlations between nucleotides at different positions, and is generally inaccurate: analysing fly and mouse *in vivo* ChIPseq data, we show that in most cases the PWM model fails to reproduce the observed statistics of TFBSs. To overcome this issue, we introduce the pairwise interaction model (PIM), a generalization of the PWM model. The model is based on the principle of maximum entropy and explicitly describes pairwise correlations between nucleotides at different positions, while being otherwise as unconstrained as possible. It is mathematically equivalent to considering a TF-DNA binding energy that depends additively on each nucleotide identity at all positions in the TFBS, like the PWM model, but also additively on pairs of nucleotides. We find that the PIM significantly improves over the PWM model, and even provides an optimal description of TFBS statistics within statistical noise. The PIM generalizes previous approaches to interdependent positions: it accounts for co-variation of two or more base pairs, and predicts secondary motifs, while outperforming multiple-motif models consisting of mixtures of PWMs. We analyse the structure of pairwise interactions between nucleotides, and find that they are sparse and dominantly located between consecutive base pairs in the flanking region of TFBS. Nonetheless, interactions between pairs of non-consecutive nucleotides are found to play a significant role in the obtained accurate description of TFBS statistics. The PIM is computationally tractable, and provides a general framework that should be useful for describing and predicting TFBSs beyond PWMs.

## Introduction

Gene regulatory networks are at the basis of our understanding of cell states and of the dynamics of their response to environmental cues. Central effectors of this regulation are Transcription Factors (TFs), which bind on short DNA regulatory sequences and interact with the transcription apparatus or with histone-modifying proteins to alter target gene expressions [1]. The determination of Transcription Factor Binding Sites (TFBSs) on a genome-wide scale is thus of central importance, and is the focus of many current experiments [2]. In eukaryotes the TF binding specificity is only moderate, meaning that a given TF may bind to a variety of different sequences *in vivo* [3]. The collection of such binding sequences is typically described by a Position Weight Matrix (PWM) which simply gives the probability that a particular base pair stands at a given position in the TFBS. The PWM provides a full statistical description of the TFBS collection when there are no correlations between the occurrences of nucleotides at different positions. In addition, the PWM description has a simple biophysical interpretation: it is exact in the special case where base-pairs of the TFBS contribute additively to the TF-DNA binding free energy at thermodynamic equilibrium, provided that the TF concentration is far from saturation [4]–[6].

Despite the widespread use and success of PWMs, there is mounting evidence that its central hypothesis of independence between positions is not always justified. Several works have reported cases of correlations between nucleotides at different positions in TFBSs [7]–[11]. A systematic *in vitro* study of 104 TFs using DNA microarrays has revealed a rich picture of binding patterns [10], including the existence of multiple motifs, strong nucleotide position interdependence, and variable spacers between determining subregions, none of which can be described by PWMs. Recently, the *in vitro* specificity of several hundred human and mouse DNA-binding domains was investigated using high-throughput SELEX. Correlations between nucleotides were found to be widespread among TFBSs and predominantly located between adjacent flanking bases in the TFBS [11]. However, TFBSs *in vivo* are certainly determined in multiple ways besides *in vitro* measured TF binding affinities and the extent of correlations between their nucleotide positions remains to be fully assessed.

On the computational side, a number of probabilistic models have been proposed to describe nucleotide correlations in TFBSs, generally based on specific simplifying assumptions, such as mutually exclusive groups of co-varying nucleotide positions [8], [12]–[14] or the computationally-friendly probabilistic structures of Bayesian networks or Markov chains [11], [15]–[17]. A systematic and general framework is yet to be applied to account for and to analyze the rich landscape of observed TF binding behaviours. The recent breakthrough in the experimental acquisition of precise, genome-wide TF-bound DNA regions with the ChIPseq technology offers the opportunity to address these two important issues.

Here, using a variety of ChIPseq experiments coming both from fly and mouse, we first show that the PWM model generally does not reproduce the observed *in vivo* TFBS statistics for a majority of TFs. This calls for a refinement of the PWM description that includes interdependence between nucleotide positions.

To this purpose, we propose the general Pairwise Interaction model (PIM), which generalizes the PWM model by accurately reproducing pairwise correlations between nucleotides in addition to position-dependent nucleotide usage. The model derives from the principle of maximum entropy, which has been recently applied with success to a variety of biological problems where correlations play an important role, from the correlated activity of neurons [18]–[21] to the statistics of protein families [22]–[26] to the alignment of large animal flocks [27]–[29]. In a thermodynamical model of TF-DNA interaction, the PIM amounts to including general effective pairwise interactions between nucleotides [30]. The PIM offers a systematic and general computational framework allowing one to determine and analyze the landscape of TF binding *in vivo*.

We find that the *in vivo* TFBS statistics and predictability are significantly improved in this refined model. We consider, for comparison, a model that describes the statistics of TFBSs as a statistical mixture of PWMs [15], [31]. This alternative model can directly capture some higher-order correlations between nucleotides, but is found to be outperformed by the PIM for all considered TFs. Further, the PIM can recapitulate the multiplicity of motifs predicted by the PWM mixture model: by studying the landscape of the PIM and its structure of local energy minima, or probability peaks, we show that each basin of attraction or valley around a local energy minimum is generally dominantly described by one PWM in the mixture model.

The difference between the PIM and the PWM model lies in the pairwise interaction between nucleotides. Surprisingly, despite significant differences in prediction accuracy between the two models, these interactions are fairly weak, sparse and found dominantly between consecutive nucleotides, in general qualitative agreement with *in vitro* binding results [11]. Comparison with a model restricted to interactions between consecutive nucleotides show that interactions between pairs of nucleotides that are farther apart nonetheless play a significant role in the prediction improvement.

The PIM only requires a modest computational effort, and the refined description of TFBS that it affords should generally prove useful when enough data is available.

## Results

### The PWM model does not reproduce the TFBS statistics

**Figure 1:**
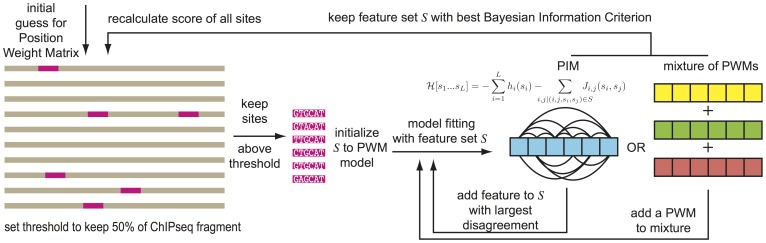
Workflow. An initial Position Weight Matrix (PWM) is used to find a set of binding sites on ChIPseq data. Models are then learned using single-point frequencies (PWM), two-point correlations (PIM) or a mixture of PWM models learned on sites clustered by K-Means with increasing complexity, *i.e.* increasing number of features in the model. Finally the models with best Bayesian Information Criteria (BIC) are used to predict new binding sites until convergence to a stable set of TFBSs.

We first tested how well the usual PWM model reproduced the observed TFBS statistics. Specifically, we asked how well the frequencies of different TFBSs were predicted using only single nucleotide frequencies. For this purpose, we used a collection of ChIPseq data available from the literature [32]–[34], both from *D. Melanogaster* and from mouse embryonic stem cells (ESC) and a myogenic cell line (C2C12). For a given TF, the ChIPseq data consist of an ensemble of DNA sequences, the ChIPseq fragments, each a few hundred nucleotides long. The exact positions of the TFBSs are unknown on each of these sequences. They are determined as 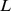-mers (we take here 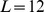) that have a score for the PWM above a given threshold score (here we chose to adjust the threshold score so that 50% of the ChIPseq fragments have at least one L-mer above the threshold score -typically, there are one or two L-mers above the threshold score on these ChIP fragments). This set of high-scoring L-mers provides a collection of putative BS s for the TF considered. From these, a PWM can be buil t in the usual way by counting the frequencies of the 4 possible nucleotide s at each position 1,…,L. However, this PWM does not coincide in general with the one that served to determine the set of putative TFBS s. To determine self-consistently the collection of binding sites for a given TF from a collection of ChIPseq fragments, we thus iteratively refined the PWM together with the set of putative TFBSs in the ChIPseq data (see Figure 1 and *Methods* for a detailed description). This process ensured that the frequency of different nucleotides at a given position in the considered set of binding sites (the high-scoring L-mers) was exactly accounted for by the PWM.

**Figure 2:**
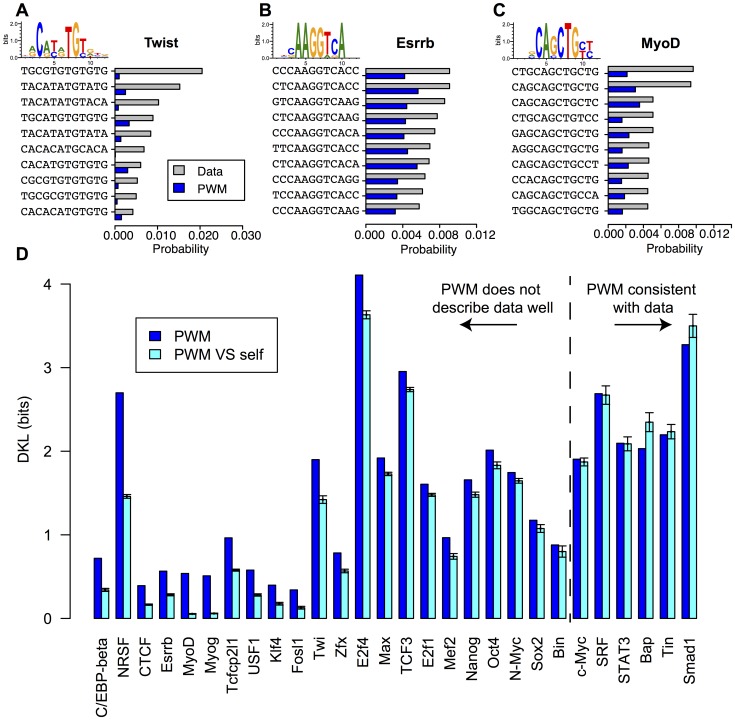
Observed TFBS frequencies are poorly predicted by a PWM model. Given a set of TFBSs predicted by the PWM model on ChIP fragments, we computed the TFBS frequencies (how many times a given sequence appears in the set, gray bars), and compared them to the PWM predicted frequencies (blue bars) computed using single nucleotide frequencies alone. We show the results for the 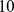 most frequent sequences for the TFs Twist (A), Esrrb (B) and MyoD (C). We can see that the use of single nucleotide frequencies alone does not allow one to reproduce the statistics of the most observed binding sites. (D) Kullback-Leibler Divergence (DKL) between the observed probability distribution and the PWM model distribution (blue). As a control we show the mean (cyan bars) along with two standard deviations of the DKL between the PWM model and a finite sample drawn from it (see Methods). A significant discrepancy between the observed and predicted sequence probabilities is reported for 22 out of 28 factors.

We then enquired whether the frequencies of the different binding sites in the set agreed with that predicted by the PWM, as would be the case if the probabilities of observing nucleotides at different positions were independent. Figure 2 shows the results for three different TFs, one from each of the three considered categories: Twist (Drosophila), Esrrb (mammals, ESC), and MyoD (mammals, C2C12). The independent PWM model strongly underestimates the probabilities of the most frequent sequences. Moreover, the PWM model does not correctly predict the frequency order of the sequences and attributes comparable probabilities to these different sequences, in disagreement with their observed frequencies.

To get a full measure of the discrepancy between the observed distribution of TFBSs and the PWM prediction, we calculated the relative entropy, or Kullback-Leibler divergence (DKL), between the two distributions [35] (see *Methods*). The DKL is zero i f the two distributions are identical and is positive otherwise. We found that for most TFs (22 out of 28), the DKL divergence was significantly larger than expected just from finite-sampling error noise (see Figure 2D). In the following, we focus on the 22 factors, for which the PWM description of the TFBSs needs to be refined. Note that the 6 factors that are satisfactorily described by the PWM model are predominantly those for which the smallest number of ChIP sequences was available (see Table 1 and Figure S1 in File S1, blue names).

**Table 1:** Information about the TFs.

Name	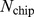	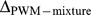	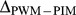	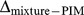	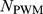	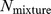	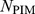
Bap	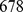	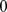	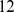	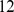	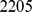	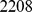	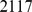
Bin	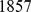	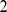	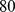	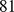	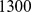	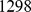	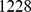
Mef2	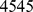	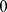	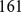	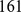	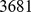	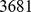	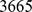
Tin	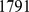	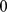	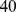	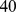	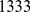	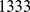	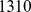
Twi	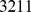	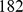	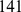	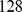	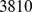	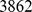	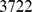
c-Myc	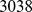	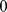	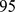	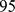	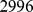	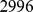	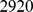
E2f1	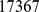	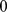	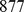	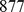	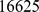	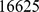	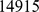
Esrrb	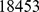	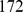	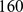	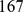	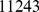	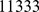	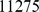
Klf4	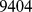	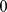	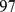	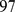	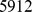	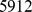	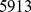
Nanog	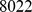	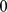	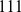	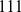	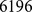	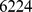	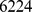
N-Myc	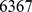	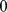	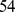	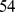	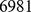	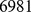	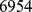
Oct4	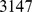	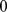	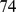	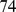	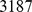	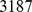	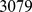
Smad1	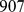	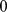	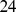	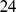	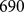	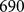	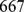
Sox2	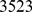	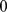	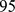	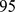	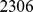	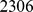	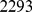
STAT3	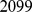	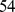	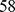	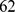	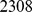	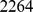	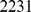
Tcfcp2l1	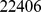	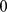	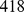	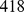	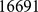	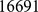	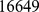
Zfx	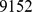	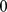	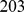	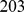	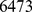	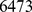	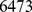
C/EBP-beta	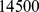	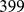	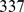	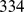	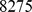	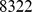	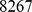
CTCF	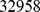	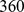	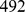	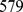	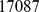	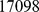	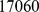
E2f4	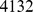	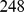	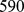	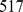	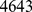	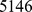	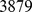
Fosl1	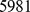	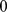	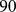	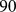	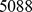	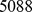	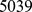
Max	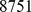	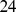	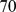	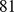	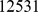	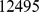	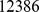
MyoD	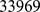	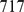	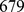	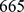	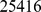	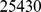	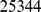
Myog	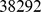	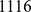	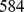	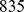	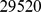	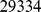	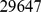
NRSF	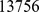	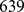	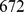	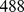	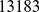	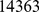	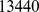
SRF	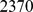	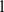	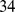	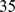	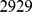	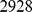	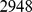
TCF3	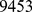	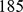	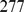	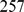	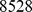	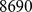	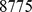
USF1	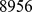	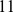	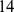	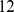	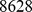	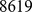	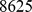

For each TF, we show the number 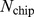 of ChIP sequences retrieved, the numbers 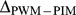, 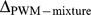, and 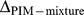 of different ChIP sequences used for training between either two models, and the numbers 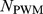, 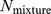, and 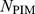 of TFBSs used to learn each model.

### Pairwise interactions in the binding energy improve the TFBS description

The discrepancy between the observed statistics of TFBSs and that predicted by the PWM model calls for a re-evaluation of the model's main hypothesis, namely the independence of bound nucleotides. To account for the correlative structure of TFBS statistics, we wish to construct a model that assigns a frequency to each of the possible 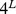 L-mers such that, given a data of observed L-nucleotide long binding sequences, the model reproduces:

- the frequency counts of the 4 nucleotides at each position in the sequence data, (e.g. 40% of nucleotides at the third position are C and 60% at the fifth position are T), as the PWM does,
- the frequency counts of each pair of nucleotides in the sequence data (e.g. 40% of pairs of nucleotides at the third and fifth position are (C,T); that is a C at the third position is always associated to a T at the fifth position and not only in 60% of the cases as would be expected for independently bound nucleotides).

There are many models that can achieve these two requirements. In order to precisely specify a single one, we ask that the model probability distribution exactly reproduces the frequency counts of single nucleotides and pairs of nucleotides in the sequence data but is otherwise as unconstrained as possible. This is the principle of maximum entropy. This provides a natural generalization of the PWM model since the PWM model is the maximum entropy model that reproduces the frequencies of single nucleotides (condition i) above). Specifically, call 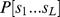 the model probability of a given TFBS sequence 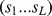. One can show (see *Methods*) that the model distribution has the following form, referred to as the Pairwise Interaction model (PIM) hereafter:
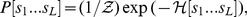
(1)



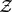
 is a normalization constant that ensures that the sum of probabilities of the different sequences is one. As an example, 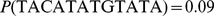 means that the model predicts that 9% of the sequences in the sequence data are the nucleotide sequence TACATATGTATA. As written under the form (1), the quantity 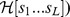 can be interpreted as the binding “energy” (in suitable units) of the considered TF for the nucleotide sequence 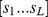. Note that with the sign convention used, the more frequent the sequence, the lower or more negative its binding energy is and the stronger its binding to the TF.

The PIM binding energy 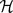 is made of two kinds of terms:
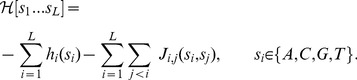
(2)


The first sum comprises the binding energies of the individual nucleotides, with 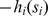 the contribution to the binding energy of nucleotide 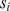 at position 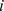. The second sum comprises the ‘pairwise interactions’, a modification of the binding energy that take s into account pairs of nucleotides in the sequence. Namely, for a sequence with a C a t the third position and a T at the fifth, the contribution of these two bases to the binding energy is not only the sum 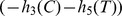 of the independent contributions but it also includes a pairwise term 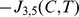.

This is an example of an inverse problem, where energies are devised from observed frequencies. As mentioned in the introduction, such problems have recently been studied in a variety of biological contexts [18]–[22], [24], [27]–[29]. In principle, the number of energy parameters in the PIM (the 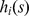
s and the 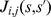 s) is sufficient to reproduce the observed values of all single nucleotide frequency counts (requirement i) above) and the frequencies of all pair s of nucleotides (requirement ii) above), that is all pairwise correlations between nucleotides at different positions (see *Methods*). However, trying to fit each of these frequencies would carry the risk of over-fitting the data with an unrealistically large number of parameters. To avoid this, we instead maximized the likelihood of the data under the model, but with a penalty proportional to the numbers of parameters involved, as provided by the Bayesian Information Criterion (BIC) [36] (see *Methods*). In addition, just as in the procedure described previously for the PWM model, the PIM and the collection of TFBSs for a given factor were iteratively refined together, as schematized in Figure 1. This is an important step to ensure that the sequence s selected by the model are identical to the sequences that serve to determine the parameters of the model.

**Figure 3:**
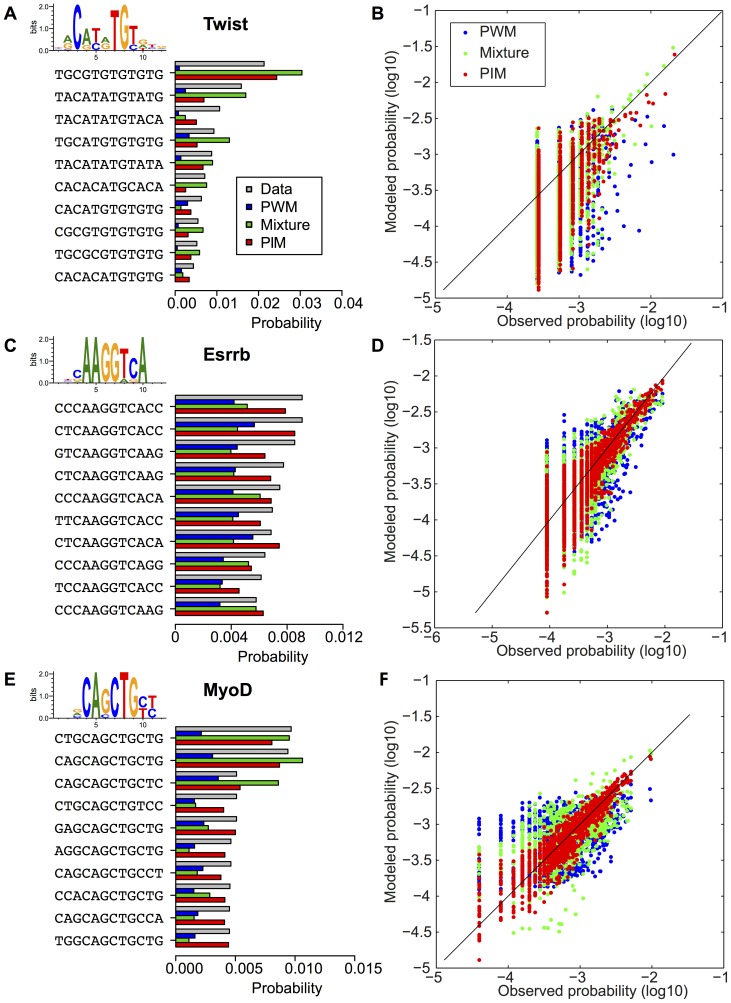
Models with correlations improve TFBS statistics prediction. Similarly to Figure 2, we show the observed frequencies (gray bars) of the most represented TFBSs for Twist (A), Esrrb (B) and MyoD (C) TFs, together with the probabilities of these sequences predicted by the PWM model (blue bars), the PIM taking into account interactions between nucleotides (red bars), and the PWM-mixture model (green bars). (B,D,F) show the comparison between frequencies for all binding sequences and predicted sequence probabilities for the three models (same color code). The predicted probabilities of the PIM and to a lesser extent of the mixture model are in much better agreement with the observed frequencies than those of the PWM model.

Figure 3 shows that the PIM greatly improves the description of TFBS statistics for the three factors chosen for illustrative purposes. Where the PWM model failed at reproducing the strong amplitude and non-linear decrease in the frequencies of the most over-represented TFBSs, the PIM provides a substantial improvement in reproducing the observed statistics. The improvement is most apparent when comparing the frequencies of the ten most observed TFBSs between the model and the ChIPseq data (Figure 3 A, C, E), and is further illustrated by the statistics of the full collection of TFBSs (Figure 3 B, D, F).

### The PIM ranks binding sites differently from the PWM model

Precise predictions of TFBSs are one important output of ChIPseq data. They condition further validation experiments such as gel mobility shift assays or mutageneses. Therefore, we assessed the difference in TFBS predictions between pairwise (PIM) and independent (PWM) models.

**Figure 4:**
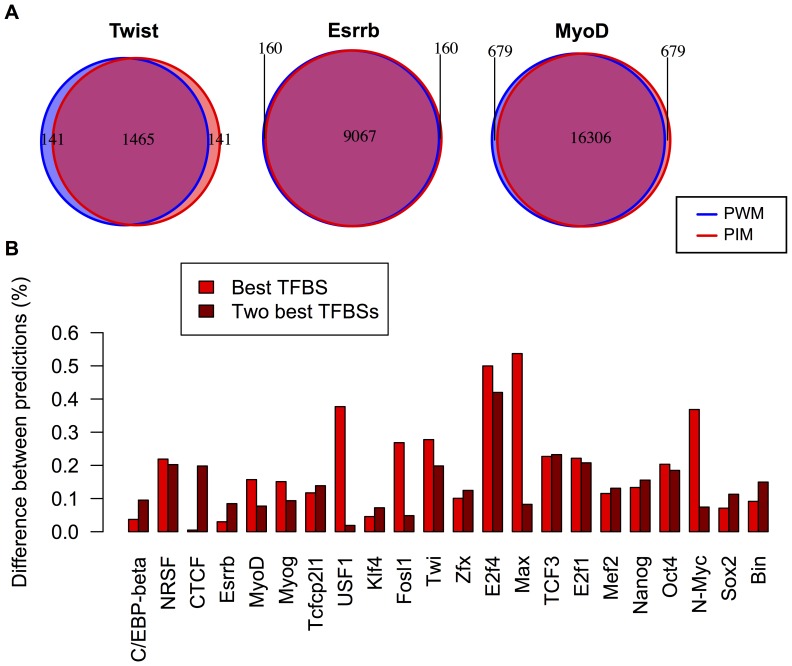
Overlap between predicted TFBSs. (A) Venn diagrams showing the overlap between the ChIP predicted by the PWM model(blue) and PIM (red). (B) Difference (one minus the proportion of shared binding sites) between the best binding sites predicted by the PIM and PWM model on ChIPseq peaks (light red), and the same quantity when including the next best predicted binding sites on each peak (dark red). In several cases (*e.g.* Fosl1, Max, N-Myc, Srf, STAT3, Usf1), the difference between predicted binding sites is much smaller when the two best binding sites are considered, indicating that the PIM and the PWM model rank differently the two best binding sites in ChIP peaks with multiple bound sites.

First, we compared the set of ChIP sequences retrieved by the two models at the cutoff of 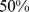 TPR (True Positive Rate) used in the learning scheme, as shown in Figure 4A. The fraction of ChIPseq sequences picked by one model but not by the other ranged from a few percent for Esrrb, up to about 15% for Twist. Thus, even when stemming from the same ChIPseq data, the two models could predict significantly distinct sets of TFBSs.

Second, using the set of ChIPseq peaks on which the PIM was learned, we looked for the best predicted binding sites on each ChIPseq bound fragment using both the PWM model and the PIM (Figure 4B and Table 1). The overlap was found to be about 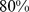 on average. We also computed the overlap between the sets comprising the two best TFBSs of each ChIPseq. When the selected fragments typically contained more than one TFBS, this resulted in an increase in overlap (*e.g* for Fosl1, Max, N-Myc, USF1). Conversely, the overlap between the models was decreased when the fragments typically contained one or less TFBS (*e.g* CTCF, Esrrb).

In conclusion, we found that the TFBS predictions made by the two models could differ significantly both in the rank of ChIPseq fragments and in the rank of binding sites on these fragments.

### Comparison with a PWM-mixture model

**Figure 5:**
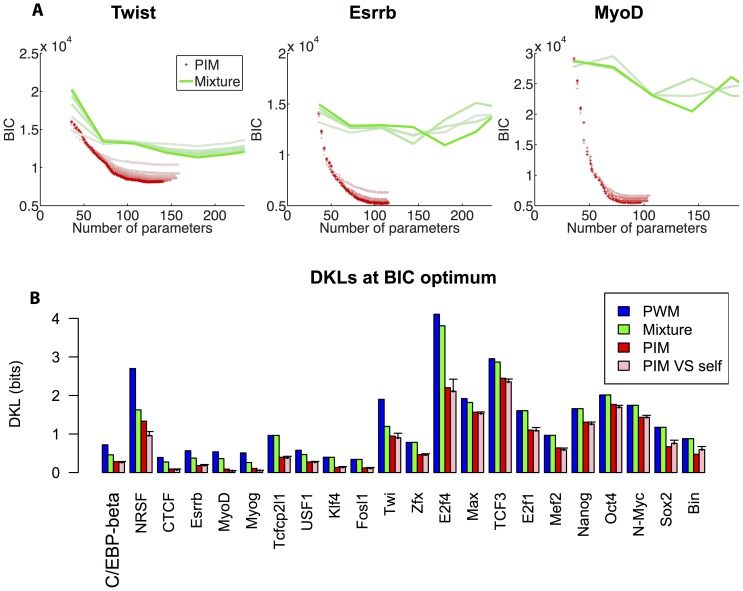
Model selection. (A) Minimisation of the Bayesian information criterion (BIC, see *Methods*) is used to select the optimal number of model parameters and avoid over-fitting the training set. The evolution of the BIC is shown for the PIM (red crosses) and the PWM-mixture model (green lines) as a function of the number of model parameters. Shades from light to dark indicate the iteration number (main loop in Figure 1), the darkest shade being assigned to the final model. (B) Kullback-Leibler divergences (DKL) between the PWM, PWM-mixture and PIM distributions and the observed distribution for the different TFs, for the BIC optimal parameters. In all cases the PIM outcompetes both the PWM and PWM-mixture models. The DKL between the PIM and a finite-size distribution of sequences drawn from it is also displayed (pink, see *Methods*) to assess the DKL magnitude simply due to the finite number of TFBS in the dataset. The result show that the PIM generally fits the available dataset as well as possible given its finite size. Error bars represent two standard deviations.

An underlying assumption of the PWM model is that there exists a preferred consensus sequence, of which other sequences are close variants. Some authors have instead analyzed the binding specificity of transcription factors by introducing multiple preferred sequences [31]. This naturally leads to a model consisting of a mixture of PWMs [15]. Such a description is a straightforward generalization of the PWM model, but potentially captures higher-order correlations than just pairwise correlations. To be able to compare with the PIM, we learned PWM-mixture models from the same ChIPseq data as previously. A mixture of 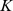 PWMs was generated by grouping TFBSs into 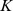 clusters. As with the PIM, the number of clusters 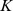 was constrained to avoid over-fitting by penalizing the model likelihood using the BIC. For a given TF, the PWM mixture and the collection of TFBSs in the ChIPseq data were refined iteratively until convergence, usually reached after 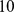 iterations (see *Methods* for details). The results are shown in Figure 5A for the three representative factors: Twist, Esrrb and MyoD.

The best description of Twi ChIPseq data is, for instance, provided by a mixture of 5 PWMs, which corresponds to 184 independent parameters. The mixture model yields a significant improvement compared to the single-PWM model, and milder ones for Essrb and MyoD. In the three cases however, it does perform as well as the PIM.

Figure 5B shows the performances of the different models for all studied TFs using the Kullback-Leibler Divergence or DKL between the observed distribution and the model distribution. The mixture model improves over the single-PWM model for 12 out of 28 TFs. The improvement is particularly good in the cases where the binding site has a palindromic structure (eg Twi, MyoD, Myog, Max, USF1). This may be explained by the fact that the TF binds DNA as a dimer, which could give some concreteness to the mixture model: the recruitment of different partners by bHLH factors like MyoD or Myog could have in fact led a mixture of TFs to bind to the set of considered binding sites. In all studied cases however, the PIM clearly outperforms the other models.

As in the PWM case, the finite size of the datasets leads us to expect fluctuations in the estimation of the DKL. In order to assess the magnitude of these finite-size fluctuations, we computed the average DKL between the best-fitting PIM and a finite-size artificial sample drawn from its own distribution, as shown in Figure 5B. Values of this DKL that are larger than the one obtained with the real dataset are indicative of overfitting, while the opposite case would suggest that the model is incomplete. In all cases, however, the DKL obtained with this control procedure was within error bars of the value computed with respect to the observed sample, with the exception of NRSF, MyoD, and Myog, as seen in Figure 5B. We thus conclude that the PIM is generally the best possible model given the available dataset.

### Multi-peaked structure of the model

The connection between the PIM and the PWM-mixture model can be further explored by considering the binding energies of all possible L-mers. This can be viewed as the “ energy landscape” of the PIM in the space of all possible binding nucleotide sequences. The “energy” of a sequence is defined in term of its probability (i.e. how frequently it appears in a set of binding sites) by 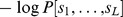 (Eq. 2) so that sequences that have a lower energy are more probable. Peaks of the probability distribution in the space of all possible L-mers therefore correspond to sequences that have a lower energy than neighboring ones. By contrast with the simple, single-minimum energy well of the PWM model corresponding to the consensus sequence, the PIM may have multiple local energy minima or probability peaks. This a general feature of of this type of models in which the parameters, the 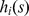
s and the 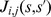 in Eq. (2), vary with position 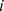, the so-called “disordered systems” [37]. Starting from any given sequence, and iteratively lowering the energy by single-nucleotide changes, one necessarily ends up in a sequence which is a local minimum. The collection of sequences falling into a particular local minimum defines its basin of attraction (see *Methods* for details). For each local minimum, we constructed a PWM model from the sequences in its basin of attraction, and associated a weight proportional to its size. We compared these PWMs to those of the mixture model, by calculating their DKL. This gave an effective distance which allowed us to associate each local minimum to the nearest PWM of the mixture model.

**Figure 6:**
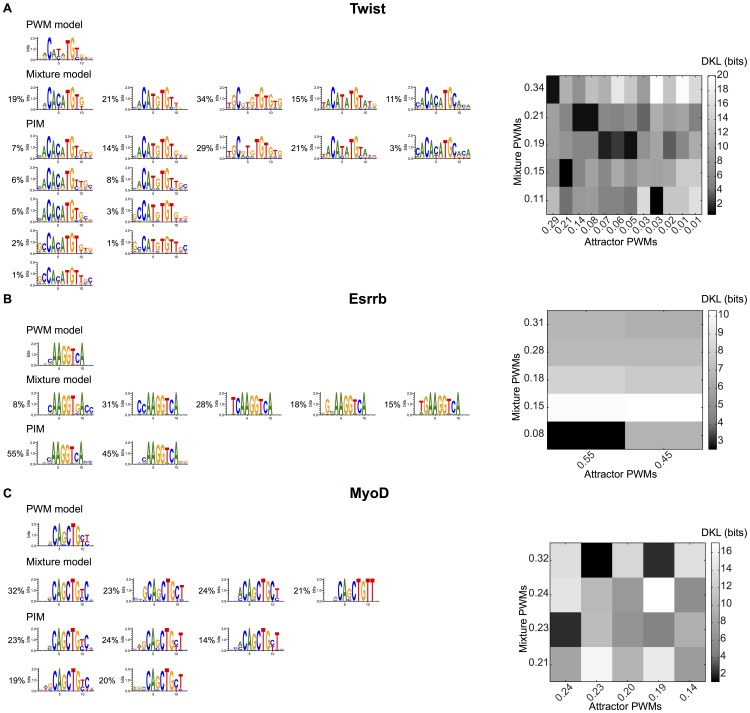
PWMs corresponding to the different basins of attraction of the PIM. The DNA sequence variety described by each model is illustrated using the software WebLogo [52]. Shown are PWMs built from all TFBS, from the PWM-mixture model, and from the basins of attraction of the PIM for Twist (A), Esrrb (B), and MyoD (C). The attractor PWMs are grouped under the mixture PWMs with smallest distance (measured by DKL, in bits). Heatmaps showing the DKLs between attractor PWMs and mixture PWMs are displayed on the right for each factor (minimal DKLs are in black). The proportions of binding sites used for each logo are also indicated and serve to denote the corresponding PWM.

Using this procedure, we computed the set of PWMs and weights corresponding to the PIM inferred from the 22 TFs for which the PWM did not offer a satisfying description. Examples are shown in Figure 6. In the case of Twist, the PWMs corresponding to different local minima, can be clearly associated to the 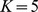 PWMs of the mixture model. For MyoD, three of the 5 PWMs based on local minima can be clearly assigned to PWMs of the mixture model. The other two have a more spread out representation. The case of Esrrb is similar: while one local minimum has a clear correspondence with a PWM of the mixture model, the other does not. The correspondence between the two models is shown in Figure S3 in File S1 for the other TFs.

This representation allows one to identify interesting features captured by the PIM. For example, in the case of Twist, most of the correlations are coming from the two nucleotides at the center of the motif, which take mainly 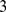 values among the 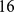 possible: CA,TG and TA. In the case of MyoD, the representation makes apparent the interdependencies between the two nucleotides following the core E-Box motif, and the restriction to the three main cases of CT, TC and TT.

### Properties of the pairwise interactions

The inference of the PIM yields explicit values for the interaction parameters 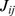, allowing for an analysis of their properties. In particular, we wondered how strong these interactions were, and how their strength depended on the distance and positions of the interacting nucleotides.

**Figure 7:**
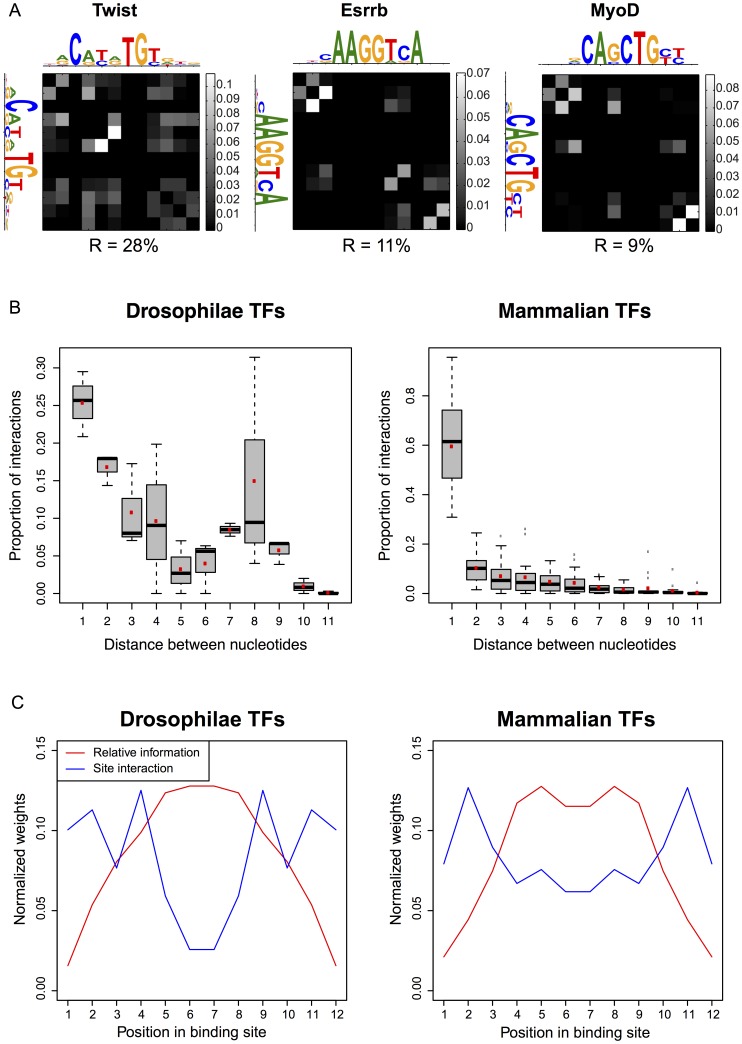
Location and strength of the nucleotide pairwise interactions. (A) Heat maps showing the values of the Normalized Direct Information between pairs of nucleotides. The matrix is symmetric by definition. PWMs are shown on the side for better visualization of the interacting nucleotides. The participation ratio R is indicated below each heat map. (B) Distances between interacting nucleotides. The box plots show the relative importance of the Normalized Direct Information as a function of the distance between interacting nucleotides. Red dots denote average values. (C) Sum of normalized direct informations in the TFBSs at a given position, averaged over all considered factors (blue line). The average site information content relative to background as a function of position is also shown (red line). In both quantities, the average over the two TFBS orientations has been taken.

To estimate the strength of interaction between two positions, we used the tool of Direct Information, originally introduced to predict contacts between residues from large-scale correlation data of protein families [38]. More specifically, we built the Normalized Direct Information (NDI), a quantity ranging from 0 for non-existing interactions, to 1 when interactions are maximum (see *Methods*). Heatmaps displaying the results for the representative Twist, Esrrb and MyoD factors are shown in Figure 7A, and in Figure S4 in File S1 for the other factors. The direct information between different nucleotides is rather weak—usually smaller than 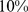
—but substantially larger than the direct interaction between nucleotides in the surrounding background (1–3%, see Figure S5 in File S1). Interestingly, such weak pairwise interactions give rise to a substantial improvement in the description of TFBS statistics, similarly to what was previously found in the neural context [18]. The pairwise interactions are concentrated on a small subset of all possible interactions. This can be made quantitative by computing the Participation Ratio of the interaction weights, an indicator of the fraction of pairwise interactions that accounts for the observed Direct Information (see *Methods*). This analysis yielded typical values of 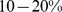 for the Participation Ratio (Figure 7A and Table 2), meaning that interactions tend to concentrate on a few nucleotide pairs. We quantified in a similar way the extent of correlations between nucleotides, using the mutual information between different positions in the TFBSs for a given factor (see *Methods*). As seen in Table 2, the pairwise interactions are found to be more sparse than the correlations, consistent with the intuition that correlations between pairs of positions in the sequences can arise indirectly from a ‘path’ of interactions, even when there is no direct interaction between nucleotides at the two positions.

**Table 2:** Participation Ratios.

Name	Part. Ratio DInorm	Part. Ratio MInorm
Bin	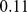	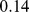
Mef2	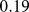	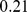
Twi	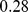	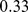
E2f1	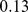	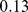
Esrrb	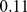	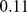
Klf4	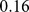	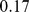
Nanog	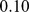	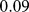
N-Myc	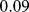	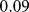
Oct4	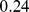	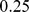
Sox2	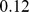	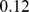
Tcfcp2l1	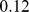	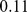
Zfx	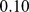	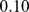
C/EBP-beta	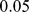	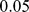
CTCF	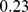	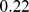
E2f4	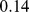	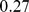
Fosl1	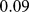	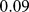
Max	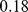	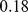
MyoD	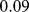	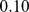
Myog	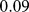	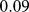
NRSF	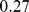	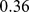
TCF3	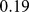	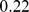
USF1	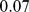	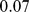

For each TF, we show the Participation Ratios computed for the Normalized Direct Information and Normalized Mutual Information matrices (see *Methods*). Interactions are generally more localized than correlations.

The interaction strength can also be used to measure the typical distance between interacting nucleotides. To that purpose, we computed the relative weight of the Direct Information as a function of the distance between nucleotides (see *Methods*). Figure 7B shows box plots summarizing the results for the considered TFs. Both plots show a clear bias towards nearest-neighbor interactions with a strong peak for nearest neighbors, and a rapid decrease for distances greater than one.

Finally, we asked how the interaction strength depended on the position along the sequence. We found that interactions were strongest in the flanking regions of the binding site, in clear anti-correlation with the information content, which concentrates in the central region (Figure 7C). These observations for TF binding *in vivo* agree with similar ones made from a large recent analysis of TF binding *in vitro* [11]. One way to rationalize them is that nucleotide diversity is required for pairwise correlations to be important.

**Figure 8:**
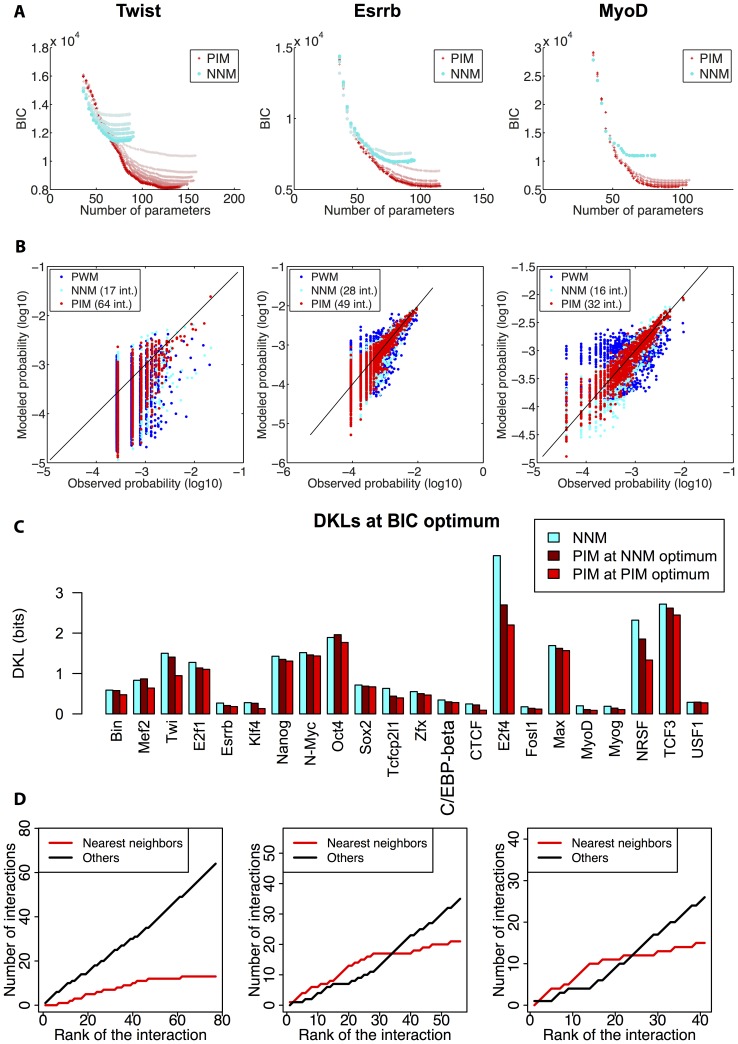
Comparison with the Nearest-Neighbor Model (NNM). We study the effect of restricting the PIM to nearest-neighbor interactions, resulting in the NNM. (A) The BIC is shown for the PIM (red crosses) and NNM (cyan dots) as a function of the number of interactions added. Shade from light to dark indicates the iteration, similarly to Figure 5. The NNM performs less well than the PIM, which provides a quantitative ground for the addition of non-neighbor interactions. (B) Comparison between the observed and predicted frequencies of TFBS according to the PWM, NNM, and PIM. We show the number of added interactions for the PIM and NNM in the legend of each plot. (C) DKLs between the NNM or PIM predicted distributions, and the observed distribution, with the number of parameters that is optimal for the NNM (first two bars) and with the number of parameters that is optimal for the PIM (last bar). The improvement yielded by the PIM is clearly seen for factors like Klf4, CTCF, E2f4 or MyoD. (D) Cumulative distribution of nearest-neighbor (red) and non nearest-neighbor (black) interactions added as a function of the number of interactions added (ranked by strength).

The fact that nearest-neighbor interactions are found to be predominant in our unbiased analysis may suggest that they are in fact sufficient to reproduce the statistics of TFBS. This appears interesting to test since PIM s restricted to nearest-neighbor interactions are equivalent to first-order Markov models which are computationally more tractable and in widespread use. In order to assess this possibility, we therefore followed the same iterative procedure as fo the PIM but only allowing the addition of nearest-neighbor interactions. The results for the resulting Nearest-Neighbor Model (NNM) are shown in Figure 8. Detailed results as a function of the number of pairwise interactions are shown for Twi, Esrrb and MyoD in Figure 8A. In the three cases, the PIM allows one to introduce more interactions than the NMM without overfitting the data, as measured by the BIC, and fits the data significantly better than the NNM (Figure 8B). Even when one restricts it self to the optimal number of interactions for the NMM, the PIM does better than the NMM.

Figure 8C more generally summarizes the results for the 22 factors, the TFBS statistics of which is not accurately described by the PWM model. While for some factors the PIM only mildly improves over the NNM in terms of DKL (e.g USF1, Sox2, N-Myc, Zfx), for many others there is a significant improvement, sometimes leading to a more than 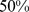 decrease of the DKL (e.g Twi, Klf4, CTCF, MyoD), when comparison is made between the two models with their optimal number of interactions (mean improvement of 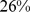, see “PIM at PIM optimum” Figure 8C). The improvement is smaller but exists for several factors even when one considers the PIM with only the small number of interactions (mean improvement of 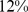) allowed to prevent overfitting for the NMM (“PIM at NMM optimum” Figure 8C). To see how this better performance of the PIM arises together with a prevalence of pairwise interactions between consecutive nucleotides, we ranked the pairwise interactions in order of decreasing strength (the absolute value of the 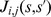) and monitored the number of interactions between consecutive nucleotides as a function of interaction strength for Twi, Esrrb and MyoD (Figure 8D). The majority of interactions between consecutive nucleotides lie among the strong pairwise interactions. Among the strong interactions, we observe a prevalence of interactions between consecutive nucleotides, especially in the case of Esrrb and MyoD. This is all the more striking that there are many more possible non nearest-neighbor interactions than nearest-neighbor ones. Yet, a number of longer range interaction are also among the strongest ones. These play a significant role, as evidenced by the better performance of the PIM over the NMM for a number of factors, even when the number of parameters in the PIM is restricted to number of interactions that is optimal for the NMM. Past the 10 to 20 strongest nearest-neighbor interactions, the number of nearest-neighbor interactions saturates and weaker interactions between distant nucleotides start to appear predominantly (Figure 8D). Although weaker, these are more numerous than the nearest-neighbor couplings and, for a number of TFs, play a significant role in improving the description of TFBS statistics (Figure 8C).

### Alternative representation of pairwise interactions by Hopfield patterns

We also analysed the interaction matrix 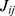 in terms of sequence-wide modes of co-variation, by diagonalizing it in an orthonormal basis of eigenvectors 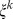, with corresponding real eigenvalues 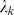. In this decomposition, the energy (Eq. (2)) can be rewritten as (see *Methods*):
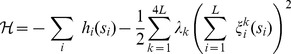
(3)


This form is reminiscent of the Hopfield model [39], which was introduced to describe neural memories as attractor patterns of the neural dynamics, arising from pairwise interactions between idealized binary neurons. Here, the role of the Hopfield patterns is played by the eigenvectors 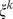. They offer an alternative way to analyze the correlative structure of the pairwise interactions, as already proposed in a mean-field context in [40], even though in this case the presence of the local binding energies 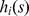, prevents the pattern sequences 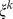 from being local energy minima in sequence space. This spectral decomposition of the interaction matrix is also similar in spirit to a principal component analysis.

**Figure 9:**
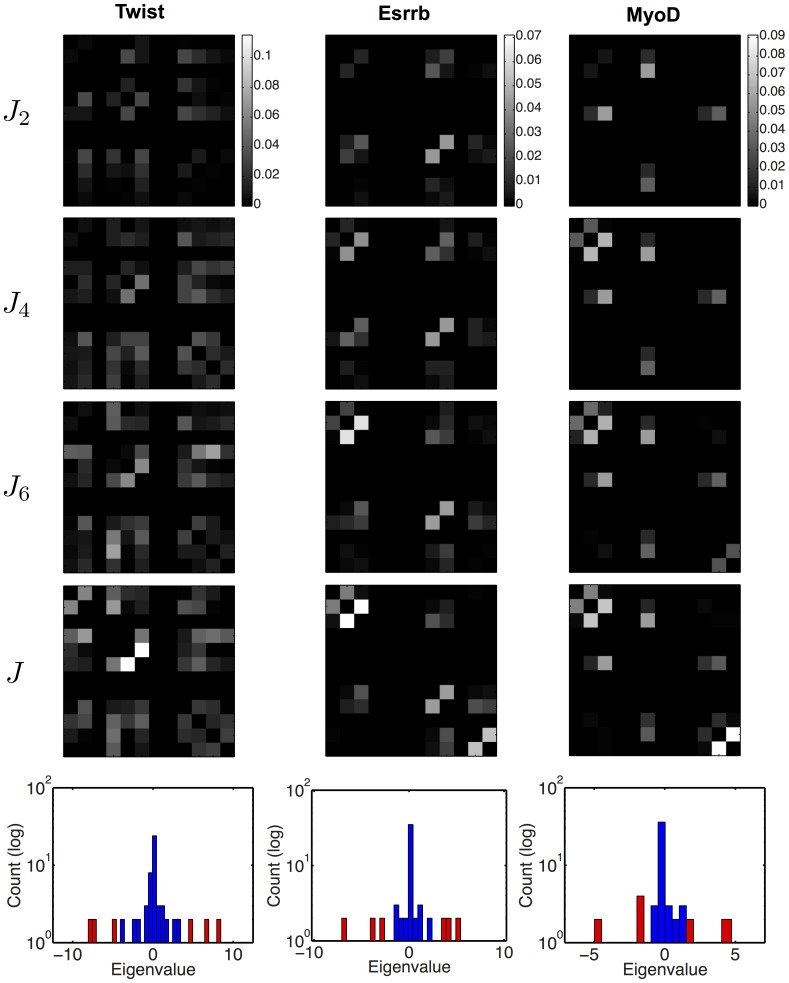
Representation of interactions by Hopfield patterns. The full interaction matrix 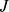 is approximated by a matrix 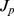 built from the 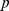 Hopfield patterns with highest eigenvalue moduli. We show the Normalized Direct Information matrices computed from 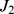, 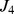, 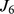 and the full matrix 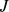. For MyoD, the correspondence between successive pairs of patterns and distinct interaction domains (middle, upper left and bottom right) is particularly clear. In all cases the full Direct Information matrix is already well approximated by 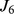. The bottom plots show histograms of the 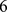 eigenvalues of highest moduli (red) and of the other ones (blue). The high eigenvalues lie on both sides of a core of smaller eigenvalues centered around 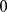.

We wondered how many patterns were necessary to approximate the full interaction matrix 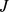 accurately. To address this question, we rank ed the eigenvalues 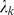 in order of decreasing moduli and noted 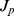 the restriction of the interaction matrix generated by the first 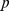 eigenvalues and their associated patterns. The full interaction matrix naturally corresponds to 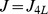. Approximate Normalized Direct Information matrices obtained by keeping increasing numbers of dominant patterns are shown in Figure 9 for the three considered representative factors. Pairs of successive patterns appear to provide the main interaction domains in this representation, as is particularly clear in the case of MyoD. One can see in Figure 9 that 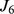 already closely approximates the full interaction matrix, a consequence of the fact that the important interactions are concentrated on a few links between pairs of nucleotides. In the eigenvalue spectrum of 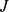, the 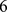 eigenvalues of highest moduli corresponding to these dominant patterns show up as ‘outliers’ as compared to the bulk of the other eigenvalues (Figure 9, red bars in the eigenvalues histograms). Theoretical analyses in the context of protein structure prediction have similarly found that large contributions to the interactions come from localized patterns with the largest eigenvalues [25], [26].

## Discussion

The availability of ChIPseq data for many TFs is an opportunity to revisit the question of nucleotide correlations in TFBSs, and to propose alternative descriptions of TFBS ensembles beyond the PWM [17]. To allow for a fair and consistent comparison between different models, we have developed a workflow in which the TFBS collection and the model describing them are simultaneously obtained and refined together. In addition, data overfitting is a concern when comparing models with many parameters, which we addressed using the BIC to penalize complex models. We found that when enough data were available, the PWM description, which assumes independence between nucleotides, failed to reproduce the observed statistics of TFBSs. The concomitant presence of correlations agrees with previous reports [7], [9], [41] and with the conclusions of large scale *in vitro* TF binding studies [10], [11].

To refine the PWM description, we have proposed and analyzed a model with general pairwise interactions (the PIM), as well as a model using a mixture of PWMs. While the mixture model somewhat improves over the PWM description, the PIM achieves a much more significant and general improvement, and can even be shown to be optimal given the amount of available data. The PIM could account for higher-order correlations than pairwise, superseding explicit descriptions in terms of multiple motifs such the one provided by the PWM-mixture model.

Several other approaches have previously been proposed to describe nucleotides correlations in TFBS, usually based on computationally-friendly approaches such as a Markov models or Bayesian networks [11], [15], [17] or focusing on strongly correlated nucleotides pairs [12]–[14]. The PIM distinguishes itself from these previous approaches by using on the contrary a ‘brute-force’ computer approach to compute the nucleotide correlations arising from the model, with the full enumeration and computation of the binding energy of all possible L-mers, without any approximation besides the limitation to pairwise interactions. This limits its application to moderate L values. The value 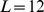 has been chosen in the present study but it could be slightly increased with more computer power or perhaps refined algorithms. With this restriction, the PIM allows one to assess the impact of simplifying assumptions. Comparison between the PIM and the NNM shows that taking into account interactions between consecutive nucleotides, as in first-order Markov models, is not sufficient to provide a good account of TFBS statistics. Similarly, we have shown that multiple weak interactions play a comparable role to the few strong correlated dinucleotides pairs [12]–[14] in improving TFBS description. Using the BIC also provides a principled way to limit the number of interactions and avoid overfitting instead of fixing a somewhat arbitrary cut-off. A study [16] proposed to improve the PWM by adding interactions describing various ‘features’ of TFBS statistics. However, this interesting proposal was only implemented in computationally simple cases (i.e for tree-like interaction structures) and it is not clear to us how it could be applied besides these. Recently a Hidden Markov Model (HMM) approach was applied to ChIPseq data, in the same spirit as was done in the present paper with the PIM [17]. The HMM approach inherits the limitations of the Markov models pointed out above, but it has the advantage that via its hidden state it can explicitly account for TFBS motifs that comprise a spacer sequence of variable length. The PIM was shown to account for higher-order correlations than pairwise, superseding explicit descriptions in terms of multiple motifs such the one provided by the PWM-mixture model. This makes it able to also account for variable length spacers, at least partially. For instance, in the case of Essrb, local probability peaks arising from nearest-neighbor interactions exhibited a triplet of flanking nucleotides with a variable spacer from the core motif (Figure S6 in File S1), possibly reflecting the inherent half-site structure of nuclear receptor binding sites. Nonetheless, extending the PIM to explicitly include the possibility of a variable length spacer within the binding motif, appears a worthy pursuit for future investigations. It would allow us to bring the full power of the PIM description to motifs for which its limit to L-mers of moderate size is really a limitation as well as to improve over the Markov model limitation of the HMM approach [17].

The PIM derives from the principle of maximum entropy, with the constraint that pairwise correlations are accurately described by the model. This approach has already been applied in a variety of biological contexts. The determination of amino acid interactions in protein structures [22], [23], [25], [26] is the closest to ours. The application differs however from ours on a technical level since the diversity of amino acids is much greater than the one of nucleotides and the lengths of proteins greatly exceed as well that of TFBSs. This renders an enumeration of possible sequences fully out of reach in the protein case. Various approximations are required to compute the model correlations and apply the maximum entropy formalism, in contrast to the present application to TFBSs. Other applications range from populations of spiking neurons [18], [19] to bird flocks [27]. As in our case, these models often have a multi-peaked probability landscape, leading to speculations about the functional interpretation of the local maxima [24], [42]. In the present case of TFBSs, local maxima may simply reflect the multiplicity of binding solutions.

The inferred parameters of the PIM provide insight into the location and strength of the effective interactions between nucleotides without potential biases coming from model simplifying assumptions. The dominant pairwise interactions are found mainly between consecutive nucleotides in the TFBS flanking regions, in agreement with *in vitro* TF binding data from extensive high-throughput SELEX experiments [11]. Our analysis also shows that nominal pairwise interactions are generally weak (at most only about 10% of the PWM weights), although they combine to yield a significant improvement in the description of the TFBS statistics through their collective effect. This is reminiscent of similar results obtained in the completely different context of correlated neuron activity [18].

However, the physical interpretation of the effective interactions is not clear, since these may combine real physical interactions with genomic correlations. This is similar to the case of protein families, where structural and functional contraints are hard to distinguish from phylogenic correlations or other observational biases [22]. Comparison between *in vitro* [10], [11] and *in vivo* binding data may help to disentangle the different possible origins of the found correlations, and seems worth pursuing. It appears similarly interesting to study how much of the found pairwise correlations can be explained on the basis of structural data. Finally, the role of nucleotide interactions in TFBS evolution [43] should be considered and could improve the reconstruction of TFBSs from multi-species comparison [44]–[46].

Independently of these future prospects, we have found that the TFBSs predicted from ChIPseq data depended significantly on the model used to extract them. Since the PIM and the developed workflow significantly improve TFBS description and require a modest computational effort, they should prove worthy tools in future data analyses.

## Methods

### Genome-wide data retrieval

We use both ChIP-on-chip data from *Drosophila Melanogaster* and ChIPseq data from *Mus Musculus*. Data was retrieved from the literature [32], [33] and from ENCODE data accessible through the UCSC website http://hgdownload.cse.ucsc.edu/goldenPath/mm9/encodeDCC/wgEncodeCaltechTfbs/, for a total of 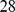 TFs. Among them, there are 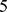 developmental Drosophilae TFs: Bap, Bin, Mef2, Tin and Twi, 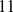 mammalian stem cells TFs: c-Myc, E2f1, Esrrb, Klf4, Nanog, N-Myc, Oct4, Sox2, STAT3, Tcfcp2l1, Zfx, and 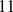 factors involved in mammalian myogenesis: C/EBP-beta, E2f4, Fosl1, Max, MyoD, Myog, NRSF, Smad1, SRF, Tcf3, Usf1. Overall, there are between 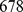 and 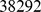 ChIP peaks, with average size 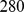
bp (see Table 1). DNA sequences were masked for repeats using RepeatMasker [47].

### Background models

It is important to discriminate the statistics of the motifs proper from that of the background DNA on which motifs are found. Besides particular nucleotides frequencies, the background DNA can exhibit significant nucleotide correlations, for instance arising from CpG depletion in mammalian genomes (Figure S5 in File S1). For each ChIPseq data, we used, as background, all 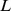-mers sequences from both strands of the ChIP peaks. This served to learn background PWM models and background PIMs which were used as reference models to score the corresponding TFBS models. The position information content in all plotted PWM logos is measured with respect to the nucleotide background frequencies (*i.e.* the PWM background model).

### Initial PWM refinement

Along with the ChIPseq data for the different factors, we also retrieved corresponding PWMs from the literature [32], from JASPAR database [48], or from TRANSFAC database version 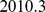 [49]. These initial PWMs were refined according to the following protocol.

First, because we restricted ourselves to binding sites of size 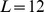 throughout this study, we sometimes had to modify the initial length of the PWMs. In those cases, we first computed the center of mass of the initial PWM column information content and used it as the new PWM center. Then we added 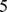 columns to the left of this center column and 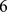 columns to the right, filling in with the initial PWM probabilities if they existed or with the background nucleotide frequencies computed on the TF ChIPseq peaks. In most cases the information content of the final PWM was close to the information content of the full length PWM (see Table S1 in File S1). Given ChIPseq data (bound regions) for a given TF and an initial PWM of length 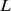 (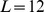 was taken for all computations in the present paper), we scanned both strands of each bound region and attributed to all observed 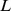-mers a score defined as the ratio between the PWM and background models probabilities. A cutoff was set such that half of the bound regions had at least one predicted TFBS with a score above the cutoff, setting a True Positive Rate (TPR) of 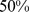. This heuristic criterion overcame the problem of False Positives among the ChIPseq peaks that might have polluted the data. This defined a training set of 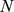 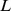-mers with probability higher than the cutoff, on which a PWM was learned. The position of the center of mass of the PWM column information content was then computed. A new PWM of length 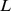 was defined centered around this position, by keeping the columns of the previous PWM that fell inside the newly defined window. The centered PWM was extended with columns of background frequencies when necessary. This ensured that the core of the motif would be found at the center, while flanking nucleotides from both sides would be represented, a feature that could be lacking from the initial PWM. Bound sites were again predicted using the same cutoff. This procedure was repeated until stabilization of the predicted sites to a fixed subset. This resulted in a refined PWM with its associated set of bound sites.

### PWM model evaluation

The PWM model consist of a matrix of single nucleotide probabilities of size 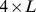, where 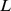 is the width of the binding site. In a first approximation, the parameters appearing in the matrix can be estimated from a set of binding sites by computing the observed frequency 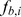 of nucleotide 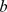 at position 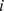. However, this frequency fluctuates around the “true” probability due to finite sample size, and for example unobserved nucleotides could actually have a low probability of being observed provided that the number of observations be high enough. It is usual to correct for this effect by using the Bayesian pseudo-count approach stemming from Laplace's rule of succession [3]. The probability to observe nucleotide 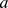 at position 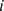 is given by:
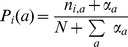
(4)where 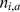 is the number of observed 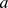 at position 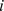, 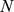 is the total number of TFBS s, and 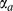
's are the pseudo-counts, or prior probabilities to observe nucleotide 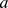 at position 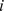. The pseudo-counts were all set to 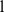, however no significant effect was noted when changing this value, as expected from the large number of observations.

### Kullback-Leibler divergence

The Kullback-Leibler divergence is a measure of distance between two probability distributions 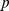 and 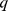 of a variable 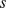, and is defined as:
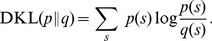
(5)


Throughout this paper, when a DKL is calculated between a finite sample and a model distribution, 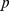 corresponds to the TFBS frequencies in the sample, and 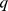 to the model distribution. When the DKL is calculated between a PWM of a basin of attraction of an attractor state and a PWM from the mixture model, 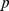 is used for the former, and 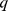 for the latter.

### Estimation of the fluctuations due to finite sampling: DKL *vs* self

To estimate whether the description of the data by a model (*e.g.* PWM or PIM) could be improved or was consistent with the finite number 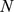 of observed sequences, we computed the ‘self’ DKL between the distribution of a set of 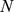 sequences drawn from the model distribution and the model distribution itself. This procedure was repeated 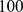 times. TFs for which the PWM model DKL was smaller than or within two standard deviations of the self DKL were discarded for later analysis.

### Derivation of the Pairwise Interaction Model (PIM)

Information theory offers a principled way to determine the probabilities of a set of states given some measurable constraints. It consists in maximizing a functional known as the entropy[50], [51] over the set of possible probability distributions given the imposed constraints. Here, we wish to determine the probability 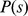 of a DNA sequence 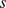 of length 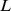, in the set of TFBSs for a transcription factor, given the constraints that the probability distribution 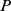 retrieves the one- and two-point correlations observed in a set of bound DNA sequences. We denote by 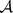 the alphabet of possible nucleotides, 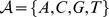 and by 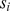 the nucleotide at position i in the sequence 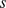 so that 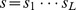. With these notations, the entropy with the considered constraints translates into the following functional:
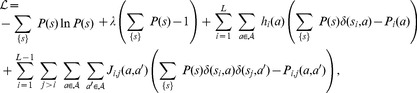
(6)where 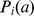 (resp. 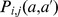) is the probability of having nucleotide 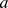 at position 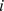 (resp. nucleotides 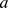 and 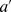 at position 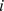 and 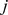) in the TFBS data set. In the evaluation of probabilities from data, pseudo-counts were set to 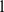 for single-point frequencies 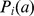 (see previous section *PWM model evaluation*) and to 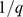 for pairwise frequencies 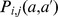, where 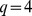 is the DNA alphabet size. The function 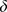 denotes the Kronecker 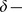
function defined by 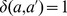 if 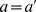,and 0 otherwise. The first term in Eq. (6) is the entropy of the probability distribution to be found and the other terms are the given constraints along with their Lagrangian multipliers. Maximization of the functional 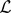 is performed in a usual way by setting the functional derivative with respect to the probability distribution 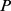 to zero:
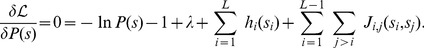
(7)


Finally, using the constraint 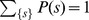, one finds the probability distribution that maximizes entropy given the constraints that it reproduces the observed one- and two-point correlations:
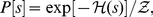
(8)where 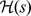 is given by,
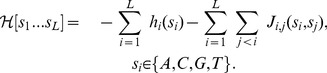
(9)


The normalization constant 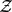 is the partition function,
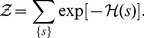
(10)


### Unique determination of the PIM

The split of the energy 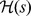 between local binding energy term and pairwise interactions is not unique and the origin of the energy scale is itself not uniquely defined. This non-uniqueness of 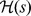 arises from the invariance of the probability distribution of sequences, as given by Eqs. (8, 9), under shifts of the local binding energies, 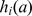 and under mutual transformations between the pairwise interaction terms 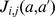 and the local binding energies. In order to uniquely determine 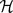, this arbitrariness needs to be taken care of by adding further conditions that uniquely fix its different energy parameters [22], a process that we detail below (this is called ‘gauge fixation’ in [22])

**Local binding energies.** The probability is invariant with respect to the following global shift of the local binding energies 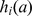, which amounts to a change of the reference energy that is cancelled by the normalization,
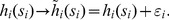
(11)


To uniquely prescribe the 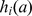, we choose to fix this invariance by minimizing the square norm 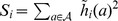 of local energy terms The corresponding condition reads
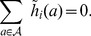
(12)


This condition can be imposed on any set of energies 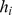 by using the tranformation (11) and redefining the energies as follows,
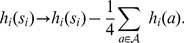
(13)


**Pairwise interactions.** Another invariance stems from the fact that contributions can be shifted between local binding energies and pairwise interactions. Namely, the following change of variables does not affect the probability:
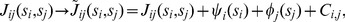
(14)since the local energy term 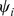 and 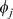 can be redistributed in 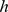 and the constant 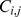 gives an energy reference for the interacting nucleotides that is cancelled by the normalization process. A unique set of pairwise interactions can be obtained by minimizing the square norm 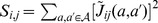 This yields the conditions:
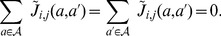
(15)


These can be imposed on any set of pairwise interactions 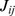 by redefining them as follows:
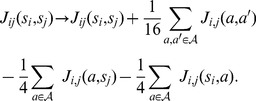
(16)


### Determination of the PIM from the data

The parameters of the model in Eq. (9), giving the energy of an observed sequence of length 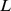, must be computed from the data. The parameters 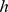 and 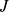 represent the energy contributions respectively coming from individual nucleotides and from their interactions. The PWM model is the particular case where all the pairwise interactions vanish: 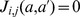.

To build the model, we start from the PWM description, characterized by the set of initial 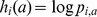 and the pairwise interactions 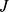
's set to zero. We add one pairwise interaction 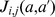 at a time, corresponding to the pair of nucleotides whose pairwise distribution predicted by the model differs most from data, as estimated by a binomial 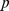-value. We then fit the augmented model to data, use this model to select a new set binding sites from the reads, and repeat the whole procedure. In each of these steps, fitting is performed by a gradient descent algorithm:
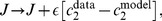
(17)

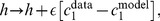
(18)where 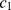 and 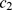 are matrices of size 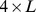 and 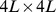 respectively corresponding to the single- and two-point frequencies, and superscripts denote whether the matrices are computed from the data or from the model distribution. This algorithm converges to the set of parameters 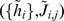 that match all single marginals and the pairwise marginals of interest. The number of pairwise interactions that are being added is controlled by the Bayesian Information Criterion, or BIC (Figure 5 in main text). The BIC computes the opposite log-likelihood and adds a penalty proportional to the number of parameters involved. This adverts the over-fitting of a finite dataset with an extravagant number of parameters. The procedure is iterated until minimization of the BIC, yielding the best PIM with the full set of parameters 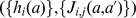. As in the case of the PWM model, we score each sequence using the ratio between the TF and background pairwise models and impose a score cutoff so as to select a set of bound sites yielding 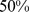 TPR, on which a new pairwise model is learned. This process is iterated until convergence to a stable set of bound sites.

### BIC computation

Consider a sample 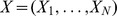 of 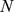 TFBSs drawn from an unknown distribution function 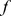 we wish to estimate. To this extent, several models 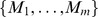 are proposed, each model 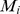 having a density 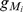 with parameter 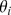 of dimension 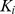. It is straightforward to see that, as 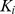 increases, the fit to the observed sample as measured by the likelihood function 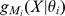 increases as well, the limiting case being when 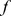 is estimated as the sample distribution. However, such an estimator is inappropriate to account for new, yet unobserved TFBSs, *i.e.* it is not predictive. Such a case where the number of parameters used to estimate a distribution becomes of the order of the size of the sample is known as overfitting. The BIC allows to overcome overfitting by penalizing high dimension parameters. Using Bayes Rule, and a uniform a priori distribution on the models, we have
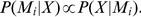
(19)


That is, the probability of the model given the data can be inferred from the probability that the data is generated by the model. The latter is obtained by marginalizing the joint distribution of the data and the parameters over the space of parameters 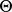:
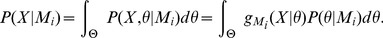
(20)


For a unidimensional parameter 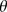, the likelihood 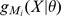 is maximized at some particular 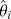 with an uncertainty (or width) proportional to 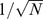 in the limit of large 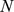. Assuming a broad prior, then for large 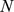 the integral is dominated by the likelihood which is concentrated around its maximum. One can then approximate the integral by the area of the region of height the maximum likelihood and of width 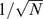, that is 
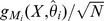
. This result can be retrieved analytically using the method of steepest descent. For a number 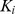 of parameters, one gets a total volume 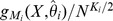 [36]. Taking the logarithm yields the BIC condition:
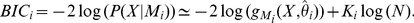
(21)


In the present case, the sample 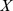 is the set of observed TFBSs and the model 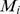 determines the probability 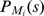 of belonging to 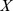,
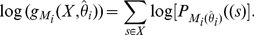
(22)


The interpretation of Eq. (21) is clear: adding new parameters improves the fit, but also adds new sources of uncertainty about these parameters due to the finite size of the data. This uncertainty disappears as 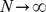, since the log-likelihood scales with 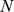 while the correction scales with 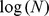.

Finally, Eq. (21) is a functional over models, the chosen model 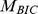 is the one that minimizes it,
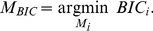
(23)


### PWM mixture model

We investigated an approach based on a mixture of PWMs. For that purpose, we used a comparable setup as for the PIM. However, instead of adding correlations to a given PWM, new PWMs were added to a mixture model. More precisely, a mixture of 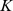 PWMs, with 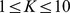, was generated by using a K-means algorithm with a Hamming distance metrics on the initial set of bound sites. This resulted in 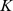 clusters, each comprising 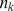 TFBSs among the initial 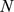 TFBSs. A PWM was generated on each of these clusters, with probability distribution 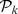. The mixture model of order 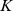 was then defined as [36]:
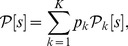
(24)where 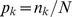 is the cluster weight. Because a PWM has 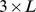 degrees of freedom (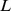 of them being constrained by the summation of nucleotide probabilities to one) and there are 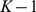 free weight parameters, the number of parameters corresponding to a mixture of order 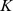 is 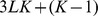. As previously, the model showing minimal BIC score was used for TFBS detection, a new set of PWMs and weights 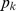 was generated by clustering the set of detected TFBS and the procedure was iterated until convergence to a stable set of TFBS.

### Local minima of the PIM and their basins of attraction

We defined the basins of attraction of a PIM energy landscape, in the following fashion. Let 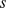 be a L-mer with energy 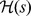. We looked for the nucleotides that could be changed to minimize 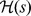. If such nucleotides existed, one of them was chosen at random, and its value was updated. One nucleotide sequence corresponding to a local energy minimum of the energy landscape, was reached when no nucleotide could be changed. The basin of attraction of a local-energy-minimum sequence was then defined as the ensemble of L-mers that fell onto this sequence when their energy was minimized. The randomness in the choice of the changed nucleotide, could in principle lead different local-energy-minimum sequences to be reached starting from the same initial sequence. However, we observed that this was rarely the case, and that results were highly similar for different runs of this procedure or when we used a deterministic method consisting in iteratively choosing the nucleotide leading to the strongest decrease in energy (Figure S2 in File S1).

We computed local-energy-minimum sequences and their basins of attraction for the final set of bound sites obtained with the best PIM. A PWM was learned on each basin of attraction, leading to a set of representative PWMs, with different weights representing different proportions of bound sites in their basins.

### Computation of the Direct Information

We wanted to build a quantity based solely on direct interactions 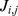 between nucleotides, discarding indirect interactions. To this end, we used the interaction parameters obtained from the PIM to build the direct dinucleotide probability function:
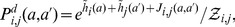
(25)where
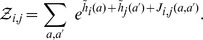



The 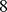 single nucleotide energies 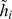 and 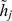 were fully determined by the constraints that the direct probability function matched the observed one-point frequencies:
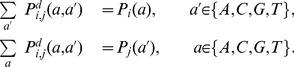
(26)


The normalization of the probabilities 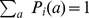, served to reduce this system to 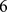 equations. The energies 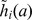, which are determined up to a constant, were fixed by the condition that they vanished for the nucleotide 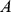, 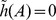. The system was solved using the Levenberg-Marquadt algorithm with 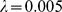. While the interaction energies (the 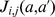
s are chosen to be those of the PIM, the single nucleotide energies 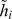 are different from the corresponding ones (the 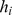) in the PIM since they originate from a different set of constraint and effectively take into account the pairwise interaction with -and between- other nucleotides.

The Direct Information [23] was then defined as:
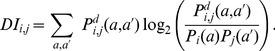
(27)


As there is no upper bound for this direct information, we built a normalized version of the direct information:
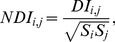
(28)where 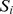 denotes the entropy at position 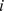. Note that 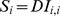 so that 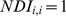 for this maximally correlated case. On the contrary, independent nucleotides give 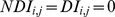.

### Computation of the Mutual Information

The Mutual Information was defined as:
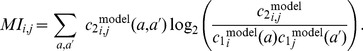
(29)where 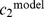 and 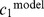 are defined in Eq. 17 and 18. As for the case of the direct information, we built a normalized version of the mutual information:
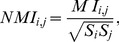
(30)where 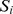 denotes the entropy at position 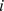.

### Participation Ratio

For each TF, an interaction weight was defined for each pair of nucleotides as
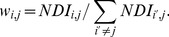
(31)


Similarly, a ‘correlation weight’ can be defined by replacing 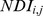 in the previous equation by 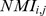. Self-interactions have no meaning here and were attributed 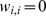. Let us note 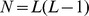 the number of possible interactions, counting both 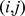 and 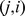 terms. Using our weight, one writes the Participation Ratio as:
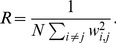
(32)


The interpretation is simple: if all weights are equal, then 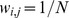 and 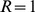, *i.e.* all possible interactions are represented. Conversely, if only one interaction accounts in the distribution budget, then 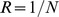, meaning that only one of all possible interactions is represented.

### Distance between interactions

The previously defined interaction weights were averaged over all possible pairs of nucleotides at a given distance 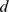 of one another, yielding the distance distribution:
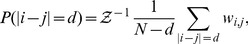
(33)


where
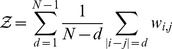
(34)is a normalization factor. Note that we introduced a correction 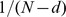 to account for finite-size effects, namely the fact that randomly distributed interactions will lead to an overrepresentation of nearest neighbors interactions just because these are more numerous.

### Interaction matrix and Hopfield patterns

In the PIM energy, shown in Eq. (1) from the main text, only 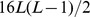 terms appear in the interaction budget: indeed, we forbid self-interactions (already accounted for by the local energy 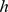) and do not count the pairwise interactions twice. However, we can straightforwardly extend the interaction matrix to a full symmetric matrix 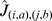 of size 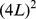, with 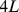-valued indices 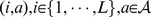. The matrix 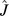 is such that for 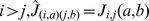 with furthermore 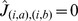 and 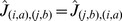. The energy of a sequence 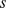 can then be written with these notations
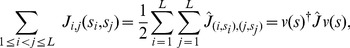
(35)where in the last equality the 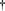 sign denotes vector transposition and we have introduced the 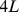 vector 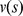 associated to sequence 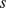, 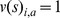 if 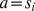 and 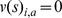 otherwise.

Since the matrix 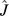 is symmetric, it can be diagonalized in an orthonormal basis of eigenvectors 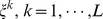 with real eigenvalues 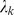,
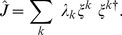
(36)


Denoting by 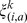 the coordinates of the k-th eigenvector then, one can rewrite Eq. (35) as
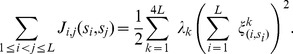
(37)


Finally, the full PIM energy is given by:
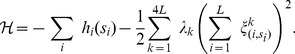
(38)


### Code availability

The source code used in the present paper is available at https://github.com/msantolini/PIM. It allows one to build the different models given sequence data (ChIP-chip or ChIPseq for example) and an initial PWM. Data files used for Twist in the present study are also provided as test examples.

## Supporting Information

File S1
**Supporting figures and tables. Figure S1. Dependence of the fit on the number of ChIP sequences.** For each TF, the number of available ChIP sequences is plotted *vs.* the improvement in the description of its TFBS statistics, provided by the he PIM as compared to the PWM model. The latter is quantified by the ratio of DKL between the respective model probability distributions and the experimental ones provided by the ChIP data, 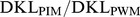. The improvement afforded by the PIM is clearly seen to be correlated to the number of ChIP sequences available.TFs for which the PWM description appears satisfactory (see Figure 2 of the main text) are shown in blue. **Figure S2. Comparison of the different methods to define the basins of attraction.** We compare two methods that allow to define the basins of attraction of the PIM model. Given an initial sequence, the attractor is found by changing iteratively either the nucleotide providing the strongest decrease in energy (deterministic method) or a random nucleotide providing a strict decrease of energy (random method). We show for the 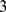 factors studied in the main text the proportion of sites falling in each of the basins of attraction using the deterministic method or 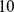 trials of the random method. For these factors we observed that the number of basins of attraction was not changing, and that the proportion of sites falling in each basin was well conserved. **Figure S3.** Same as Figure 6 of the main text for all considered factors described by a mixture model with two or more PWMs. **Figure S4.** Same as Figure 7A of the main text for the other considered factors. **Figure S5. Background correlations.** (A,B,C) Heat maps showing the correlations between nucleotides in the ChIP data of the 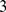 factors from the main text. Because of translation invariance, we only show the correlations between a nucleotide (rows) and the next nearest (first four columns) to farthest (last four columns) nucleotides, using the binding site length of 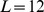. We see in the Drosophila data the appreciable presence of repeated sequences (of type AA, TT, CC, and GG). In the mammalian data sets, we observe the known CpG depletion. (A′,B′,C′) Corresponding heat maps showing the values of the Normalized Direct Information between pairs of nucleotides. **Figure S6. Variable spacer length.** We learned a PIM for Esrrb including the 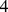 flanking nucleotides on the left of the main motif. (A) The metastable states of this model show a feature not captured in the main text where binding sites are defined symmetrically around the center of mass of the information content: namely a ‘CAG’ trinucleotide with variable spacer length from the main motif. This feature is apparent in the first 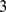 logos shown here. (B) The contribution of this trinucleotidic interaction to the Direct Information is captured through strong direct links between the 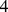 flanking nucleotides, showing that the PIM is implicitly able to capture higher order correlations. Logos from the PWM model are surrounding the heatmap for clarity. **Table S1. Comparison between initial PWMs and** 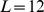 **PWMs.** Bottom rows correspond to the 6 factors that are satisfactorily described by the PWM model. Information content is in bits.(PDF)
