# Supplementary material for: A General Pairwise Interaction Model Provides an Accurate Description of In Vivo Transcription Factor Binding Sites
Source: PLoS One. 2014 Jun 13;9(6):e99015. doi: 10.1371/journal.pone.0099015 (PMC4057186; doi:10.1371/journal.pone.0099015)
Supplement: File S1 — Supporting figures and tables. Figure S1. Dependence of the fit on the number of ChIP sequences. For each TF, the number of available ChIP sequences is plotted vs. the improvement in the description of its TFBS statistics, provided by the he PIM as compared to the PWM model. The latter is quantified by the ratio of DKL between the respective model probability distributions and the experimental ones provided by the ChIP data, . The improvement afforded by the PIM is clearly seen to be correlated to the number of ChIP sequences available.TFs for which the PWM description appears satisfactory (see Figure 2 of the main text) are shown in blue. Figure S2. Comparison of the different methods to define the basins of attraction. We compare two methods that allow to define the basins of attraction of the PIM model. Given an initial sequence, the attractor is found by changing iteratively either the nucleotide providing the strongest decrease in energy (deterministic method) or a random nucleotide providing a strict decrease of energy (random method). We show for the factors studied in the main text the proportion of sites falling in each of the basins of attraction using the deterministic method or trials of the random method. For these factors we observed that the number of basins of attraction was not changing, and that the proportion of sites falling in each basin was well conserved. Figure S3. Same as Figure 6 of the main text for all considered factors described by a mixture model with two or more PWMs. Figure S4. Same as Figure 7A of the main text for the other considered factors. Figure S5. Background correlations. (A,B,C) Heat maps showing the correlations between nucleotides in the ChIP data of the factors from the main text. Because of translation invariance, we only show the correlations between a nucleotide (rows) and the next nearest (first four columns) to farthest (last four columns) nucleotides, using the binding site length of . We see in the Drosophila data [file pone.0099015.s001.pdf]

A General Pairwise Interaction Model Provides an Accurate Description of *In Vivo* Transcription Factor Binding Sites

Marc Santolini, Thierry Mora, Vincent Hakim*
Laboratoire de Physique Statistique, CNRS, Université P. et M. Curie, Université D. Diderot, École Normale Supérieure, Paris , France.
∗ Corresponding Author : hakim@lps.ens.fr
April 16, 2014

Supporting Figures

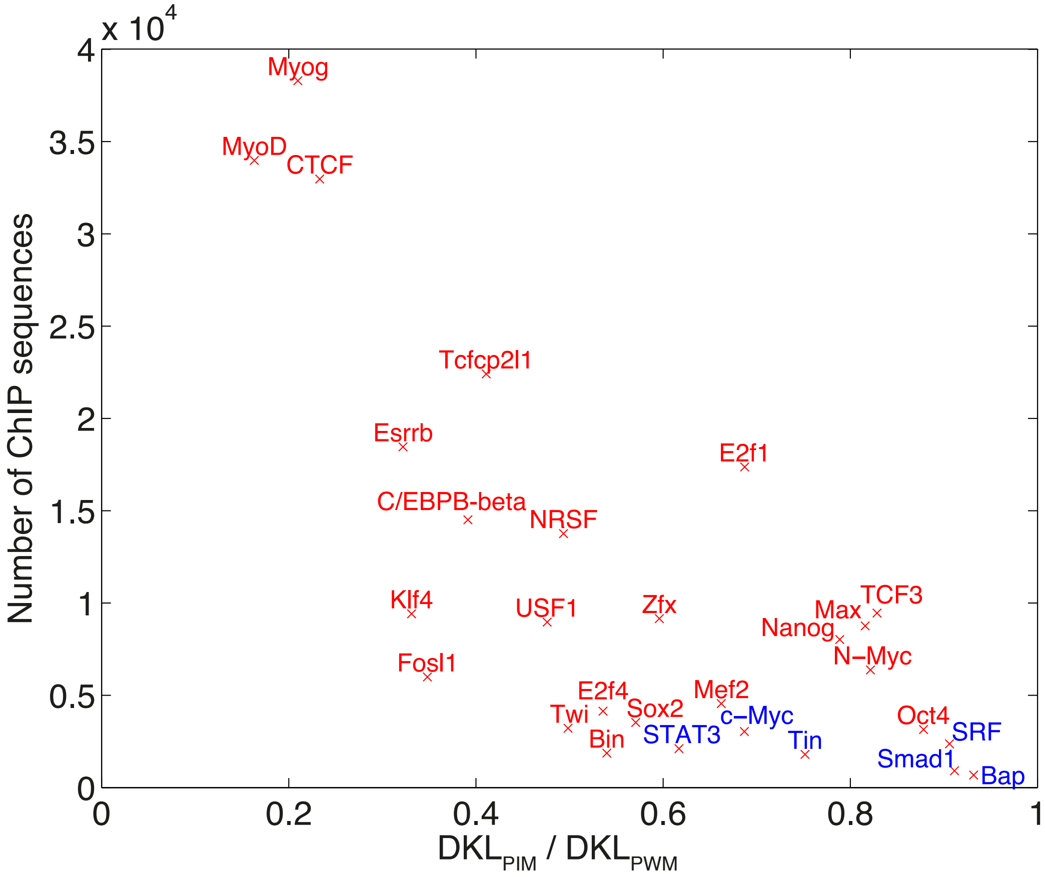

Figure S1. Dependence of the fit on the number of ChIP sequences. For each TF, the number of available ChIP sequences is plotted *vs.* the improvement in the description of its TFBS statistics, provided by the he PIM as compared to the PWM model. The latter is quantified by the ratio of DKL between the respective model probability distributions and the experimental ones provided by the ChIP data, DKL_{PIM}/DKL_{PWM}. The improvement afforded by the PIM is clearly seen to be correlated to the number of ChIP sequences available.TFs for which the PWM description appears satisfactory (see Figure 2 of the main text) are shown in blue.

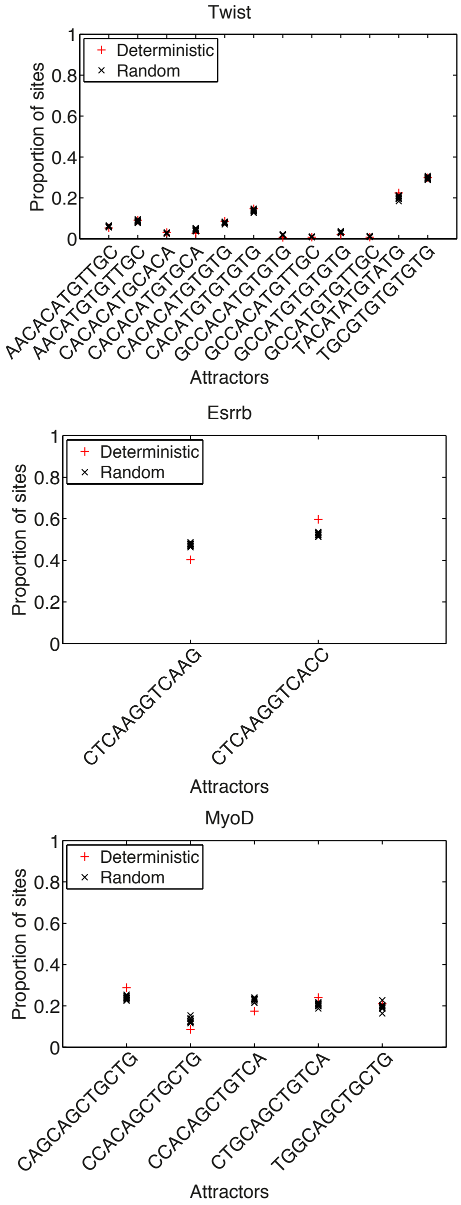

Figure S2. Comparison of the different methods to define the basins of attraction. We compare two methods that allow to define the basins of attraction of the PIM model. Given an initial sequence, the attractor is found by changing iteratively either the nucleotide providing the strongest decrease in energy (deterministic method) or a random nucleotide providing a strict decrease of energy (random method). We show for the 3 factors studied in the main text the proportion of sites falling in each of the basins of attraction using the deterministic method or 10 trials of the random method. For these factors we observed that the number of basins of attraction was not changing, and that the proportion of sites falling in each basin was well conserved.

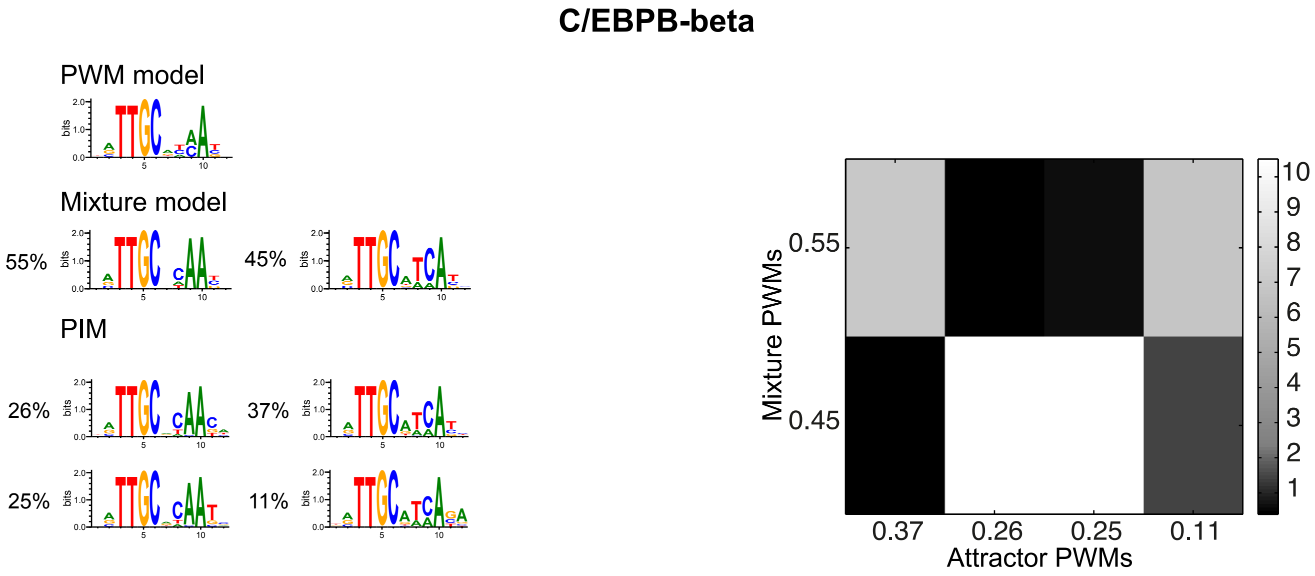
C/EBPB-beta
PWM model
Mixture model
55%
45%
PIM
26%
37%
25%
11%
Mixture PWMs
0.55
0.45
0.37
0.26
0.25
0.11
Attractor PWMs
10
9
8
7
6
5
4
3
2
1

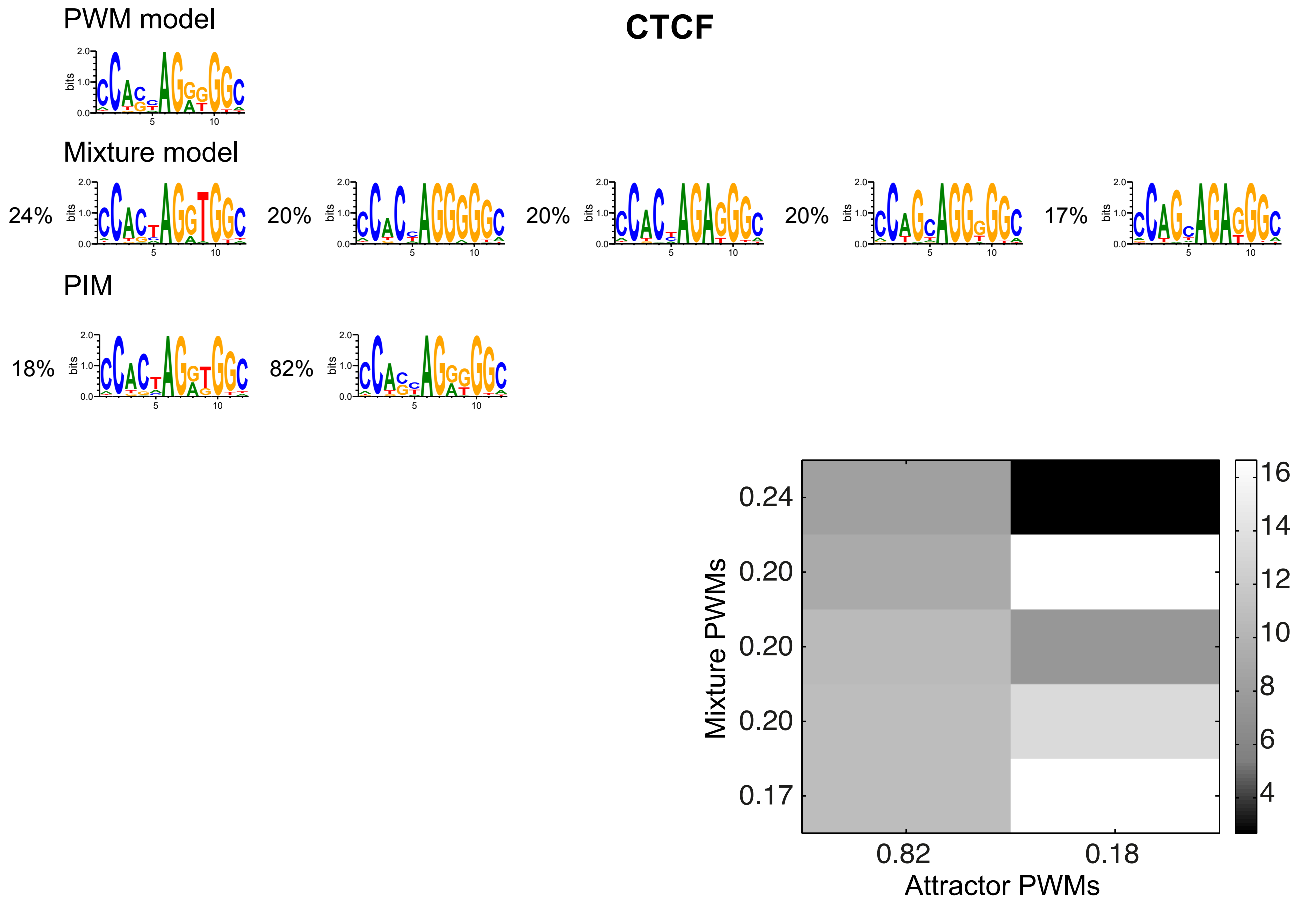
CTCF
PWM model
Mixture model
24%
20%
20%
20%
17%
PIM
18%
82%
Mixture PWMs
0.24
0.20
0.20
0.20
0.17
0.82
0.18
Attractor PWMs
16
14
12
10
8
6
4

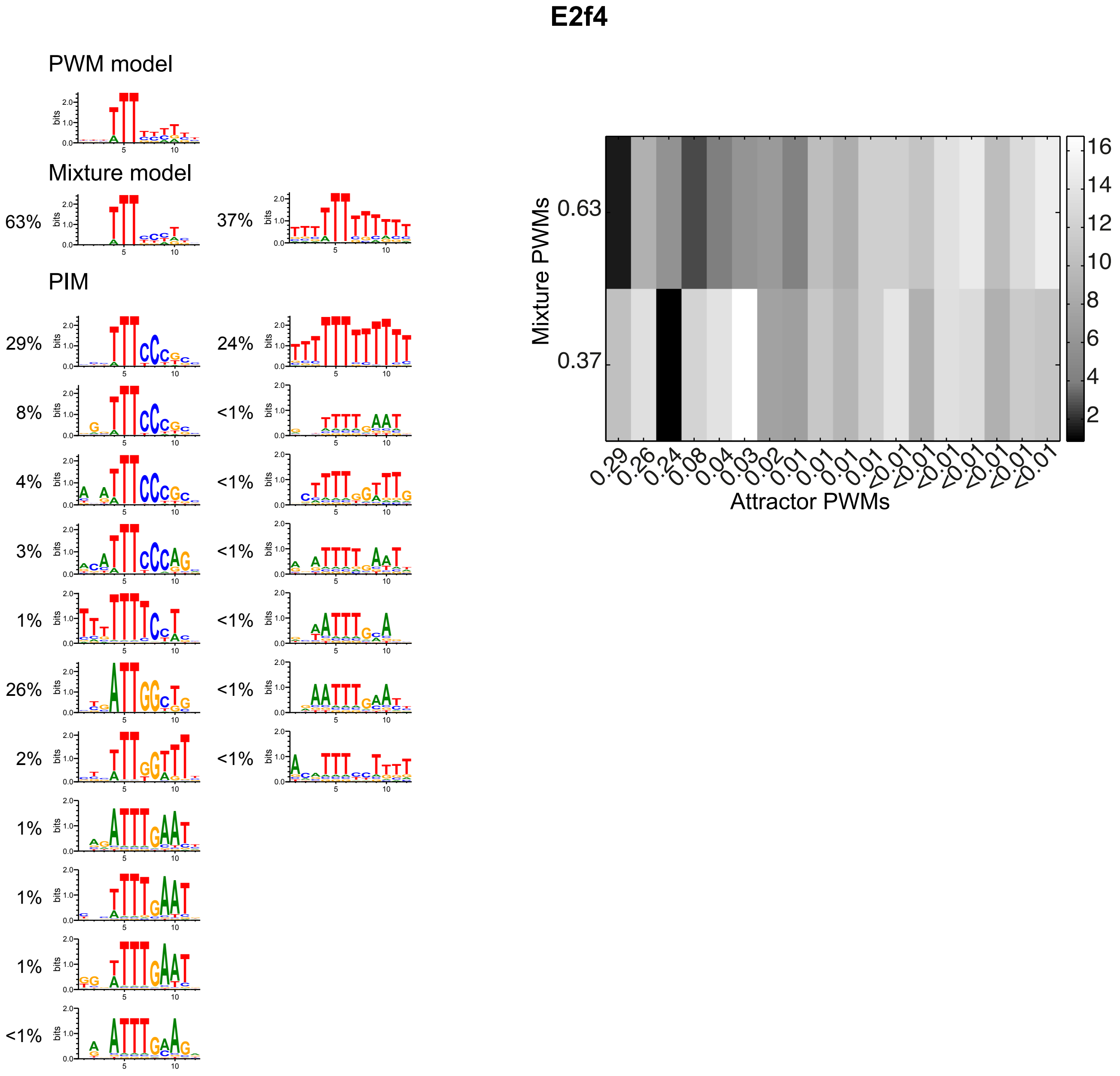
E2f4
PWM model
Mixture model
63%
37%
PIM
29%
24%
8%
<1%
4%
<1%
3%
<1%
1%
<1%
26%
<1%
2%
<1%
1%
1%
1%
<1%
Mixture PWMs
0.63
0.37
16
14
12
10
8
6
4
2
0.29
0.26
0.24
0.08
0.04
0.03
0.02
0.01
0.01
0.01
0.01
<0.01
<0.01
<0.01
<0.01
<0.01
<0.01
<0.01
Attractor PWMs

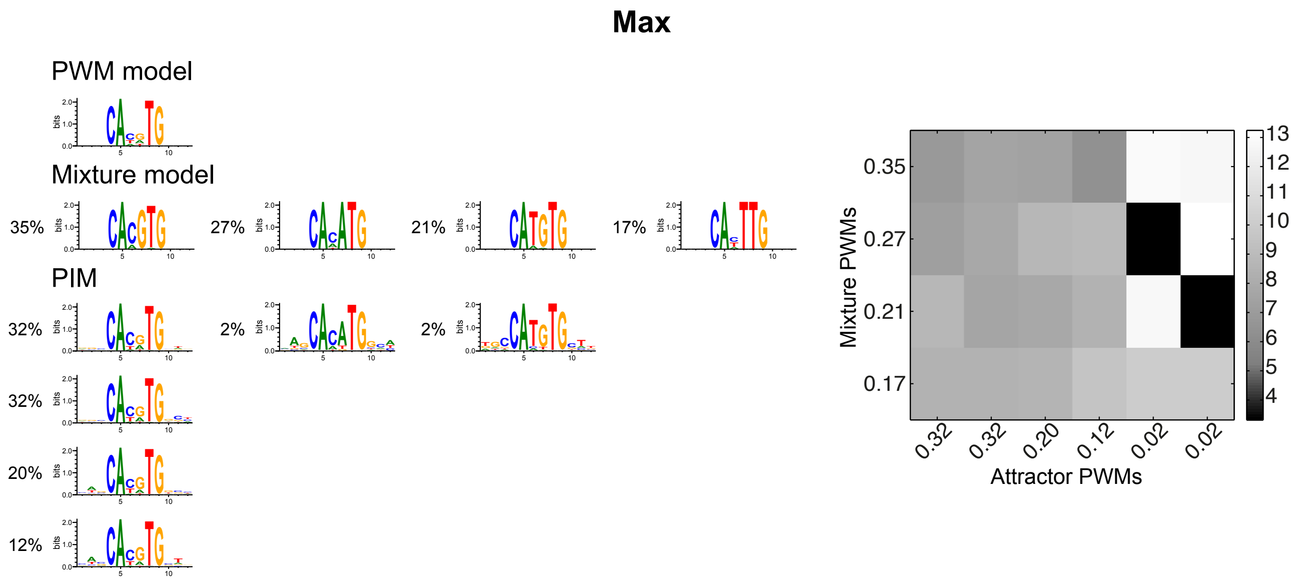
Max
PWM model
Mixture model
35%
27%
21%
17%
PIM
32%
2%
2%
32%
20%
12%
Mixture PWMs
0.35
0.27
0.21
0.17
Attractor PWMs
0.32
0.32
0.20
0.12
0.02
0.02
13
12
11
10
9
8
7
6
5
4

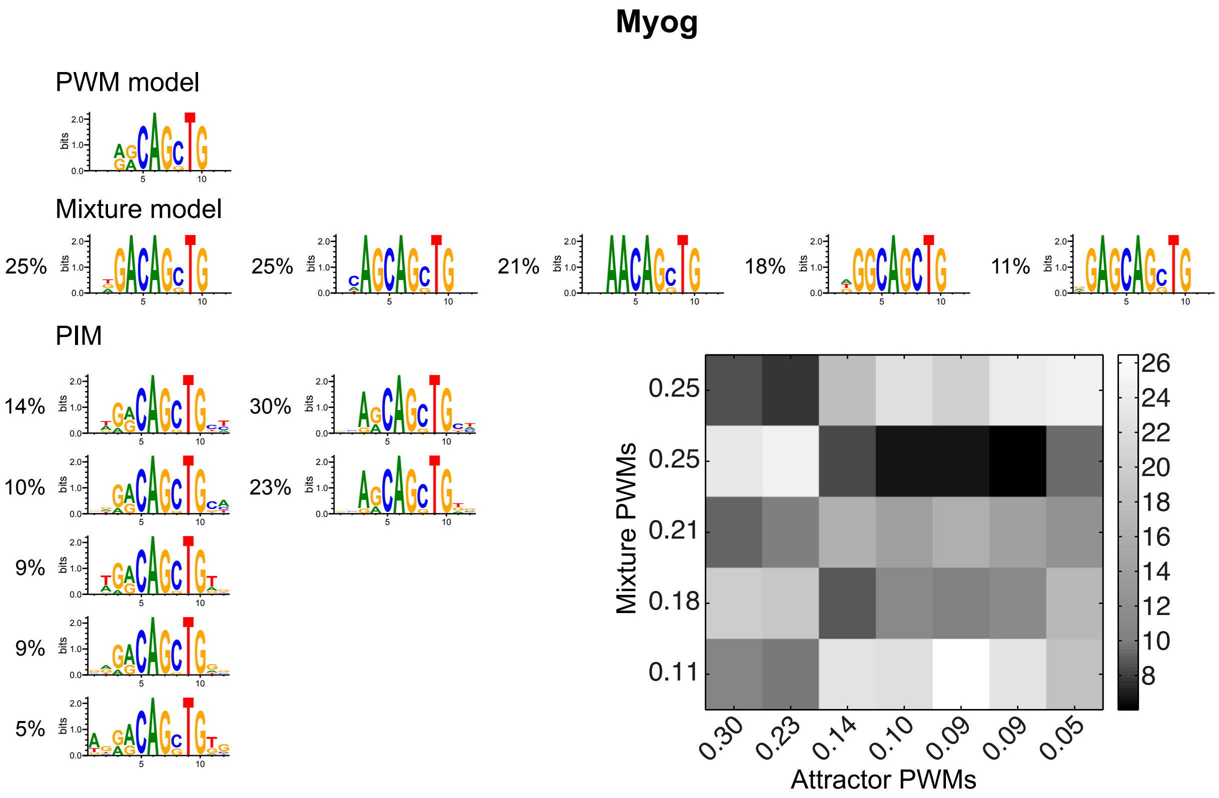
Myog
PWM model
Mixture model
25%
25%
21%
18%
11%
PIM
14%
30%
10%
23%
9%
9%
5%
Mixture PWMs
0.25
0.25
0.21
0.18
0.11
Attractor PWMs
0.30
0.23
0.14
0.10
0.09
0.09
0.05
26
24
22
20
18
16
14
12
10
8

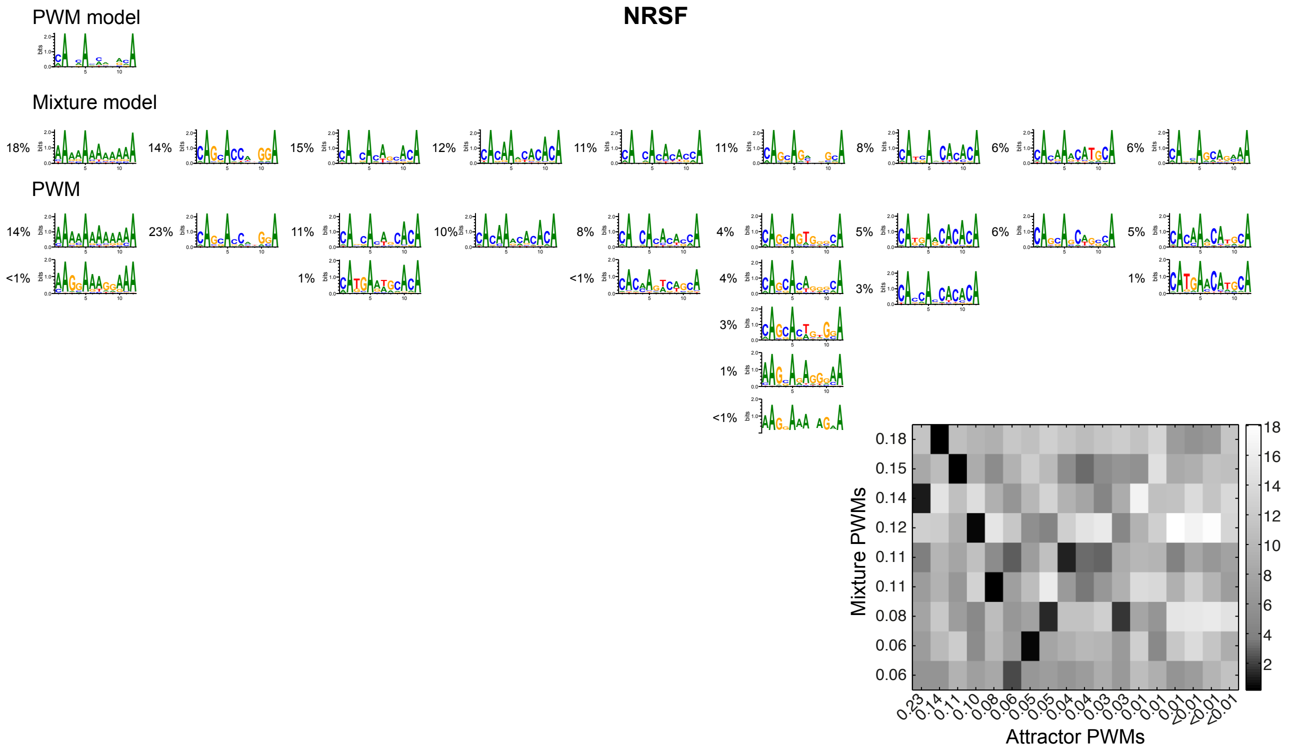
NRSF
PWM model
Mixture model
18%
14%
15%
12%
11%
11%
8%
6%
6%
PWM
14%
23%
11%
10%
8%
4%
5%
6%
5%
<1%
1%
<1%
4%
3%
1%
3%
1%
<1%
Mixture PWMs
Attractor PWMs
0.18
0.15
0.14
0.12
0.11
0.11
0.08
0.06
0.06
0.23
0.14
0.11
0.10
0.08
0.06
0.05
0.05
0.04
0.04
0.03
0.03
0.01
0.01
0.01
<0.01
<0.01
<0.01
18
16
14
12
10
8
6
4
2

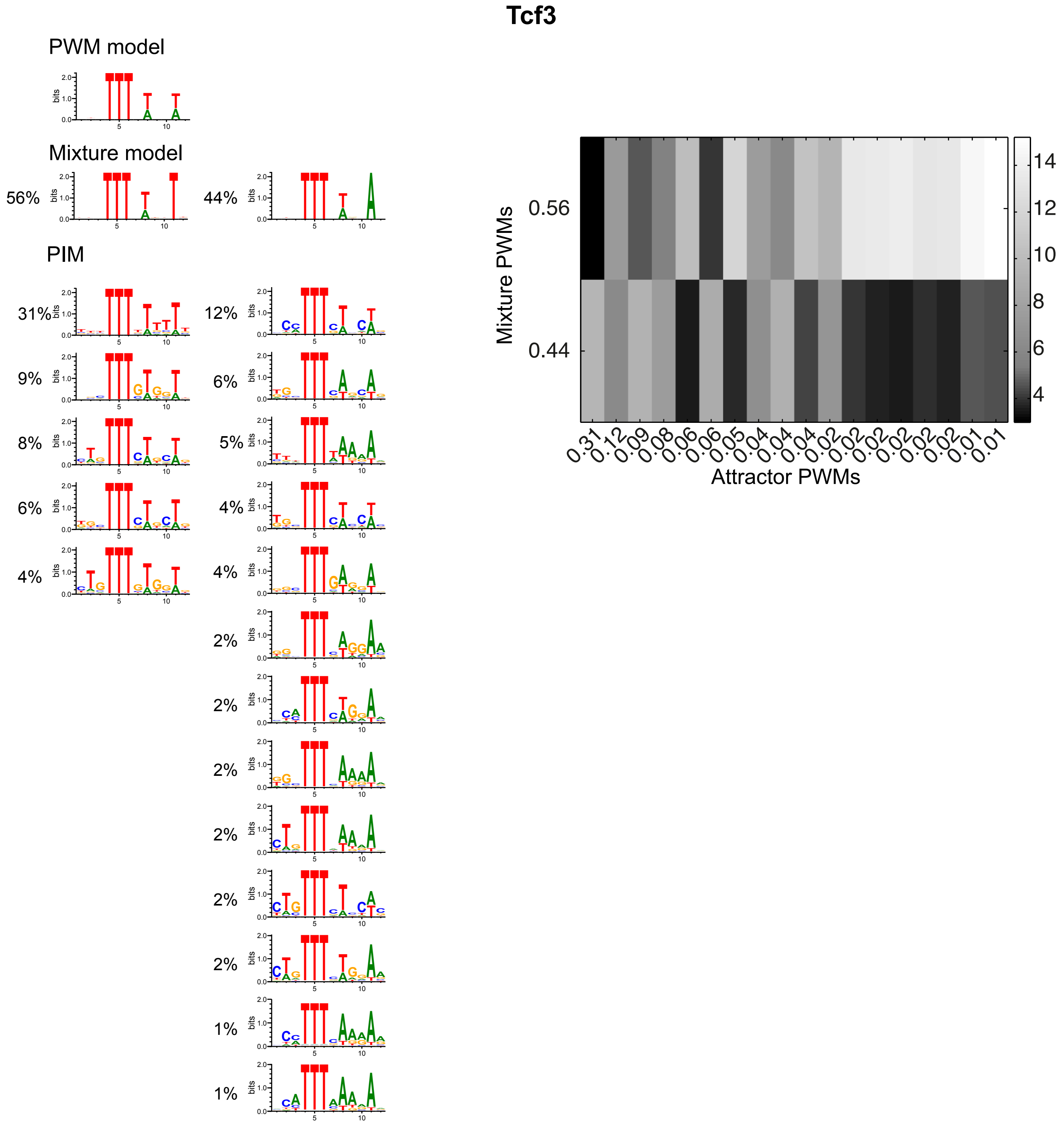
Tcf3
PWM model
Mixture model
56%
44%
PIM
31%
12%
9%
6%
8%
5%
6%
4%
4%
4%
2%
2%
2%
2%
2%
2%
1%
1%
Mixture PWMs
0.56
0.44
Attractor PWMs
0.31 0.12 0.09 0.08 0.06 0.06 0.05 0.04 0.04 0.04 0.02 0.02 0.02 0.02 0.02 0.02 0.01 0.01
14
12
10
8
6
4

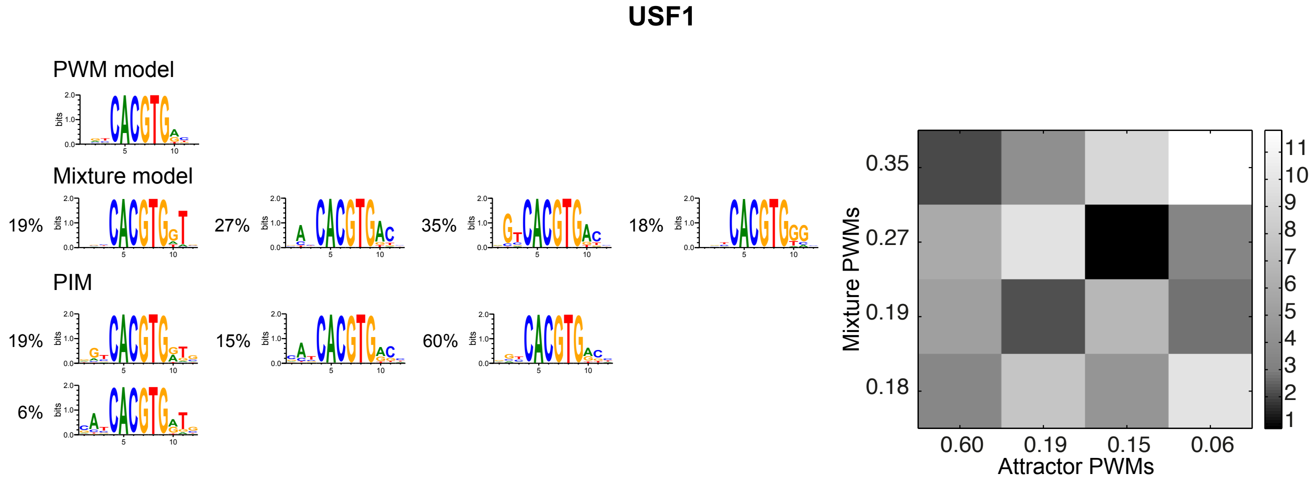

Figure S3. Same as Figure 6 of the main text for all considered factors described by a mixture model with two or more PWMs.

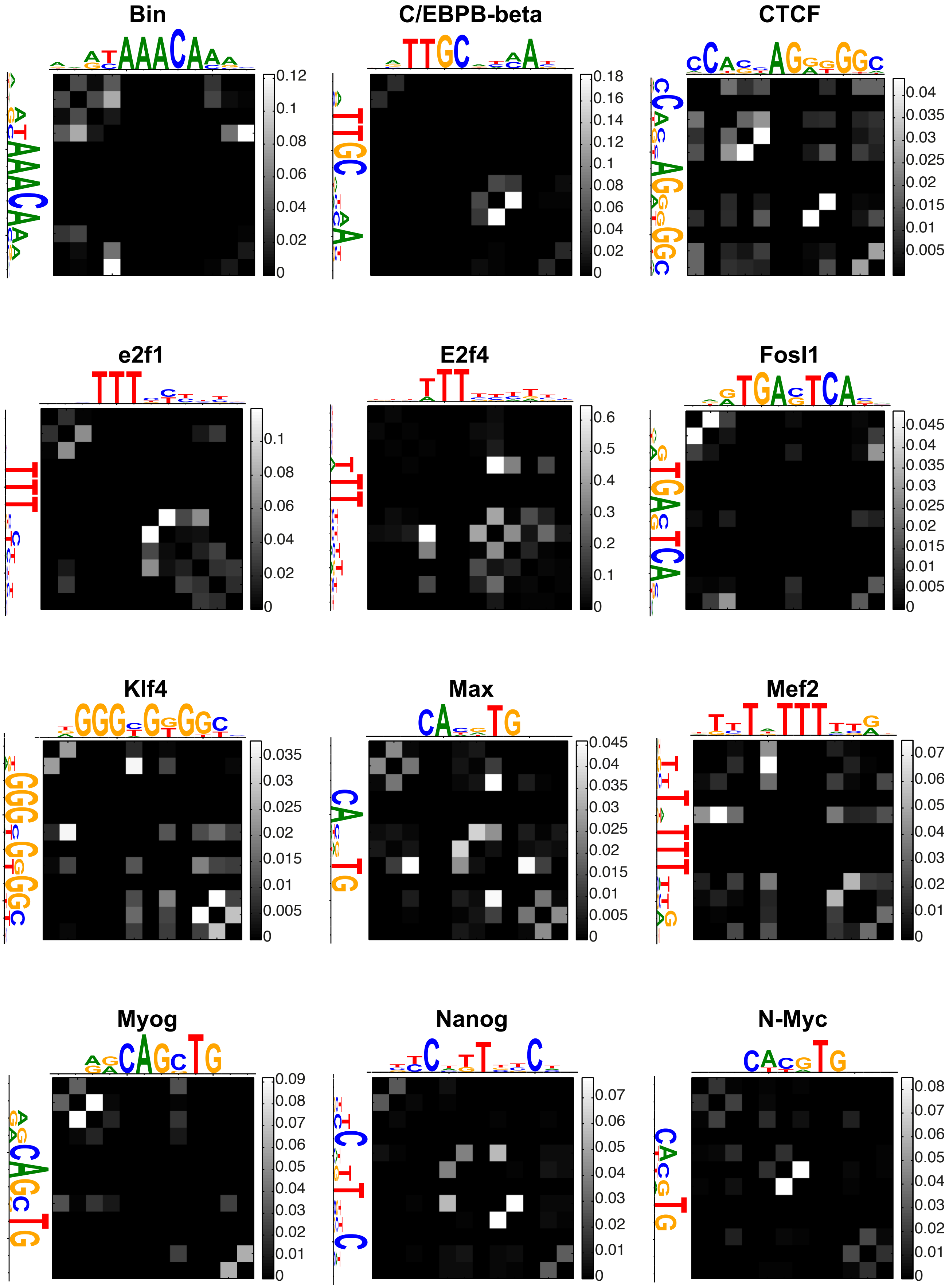
Bin
0.12
0.1
0.08
0.06
0.04
0.02
0
C/EBPB-beta
0.18
0.16
0.14
0.12
0.1
0.08
0.06
0.04
0.02
0
CTCF
0.04
0.035
0.03
0.025
0.02
0.015
0.01
0.005
e2f1
0.1
0.08
0.06
0.04
0.02
0
E2f4
0.6
0.5
0.4
0.3
0.2
0.1
0
Fosl1
0.045
0.04
0.035
0.03
0.025
0.02
0.015
0.01
0.005
0
Klf4
0.035
0.03
0.025
0.02
0.015
0.01
0.005
0
Max
0.045
0.04
0.035
0.03
0.025
0.02
0.015
0.01
0.005
0
Mef2
0.07
0.06
0.05
0.04
0.03
0.02
0.01
0
Myog
0.09
0.08
0.07
0.06
0.05
0.04
0.03
0.02
0.01
0
Nanog
0.07
0.06
0.05
0.04
0.03
0.02
0.01
0
N-Myc
0.08
0.07
0.06
0.05
0.04
0.03
0.02
0.01
0

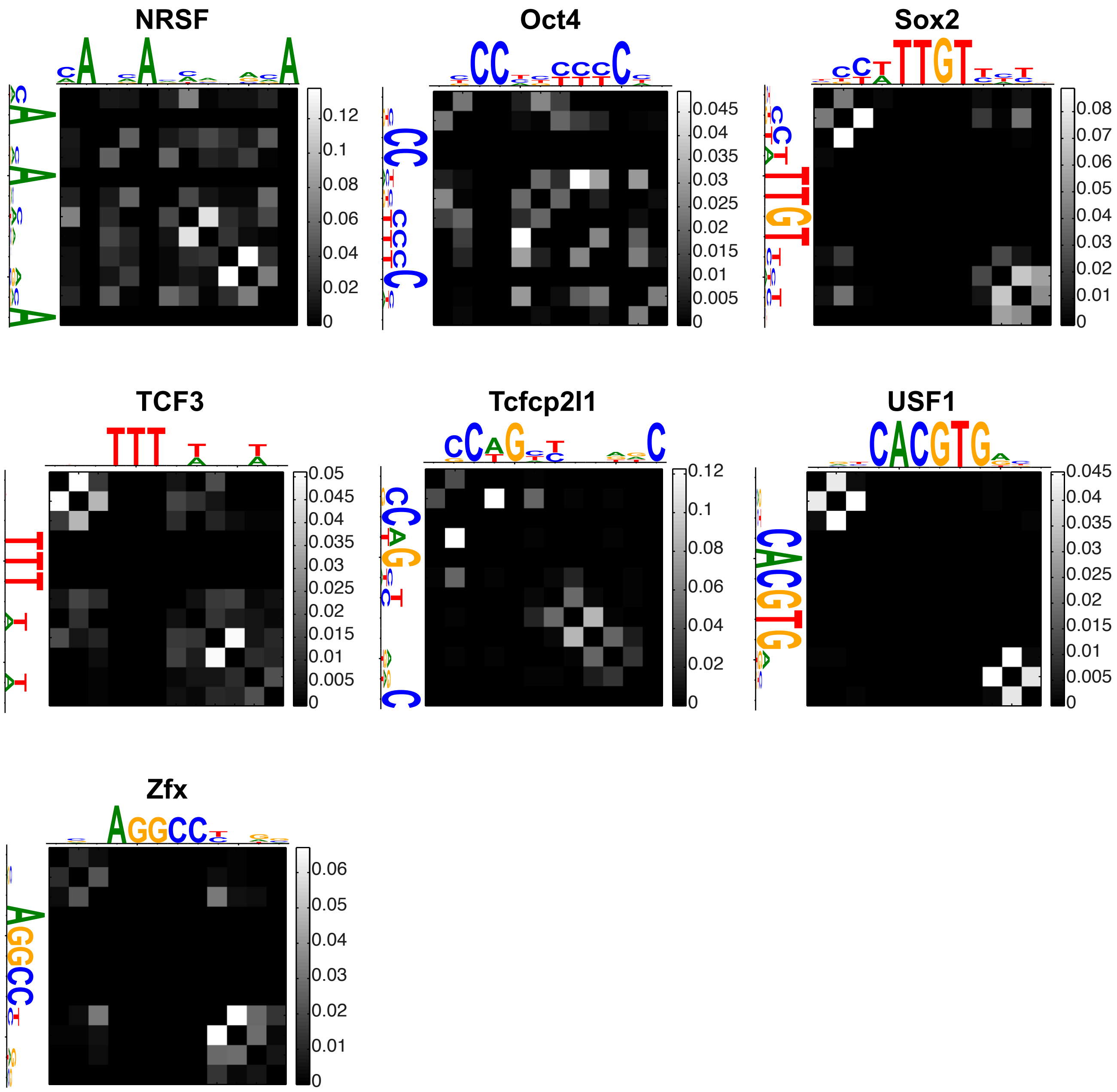

Figure S4. Same as Figure 7A of the main text for the other considered factors.

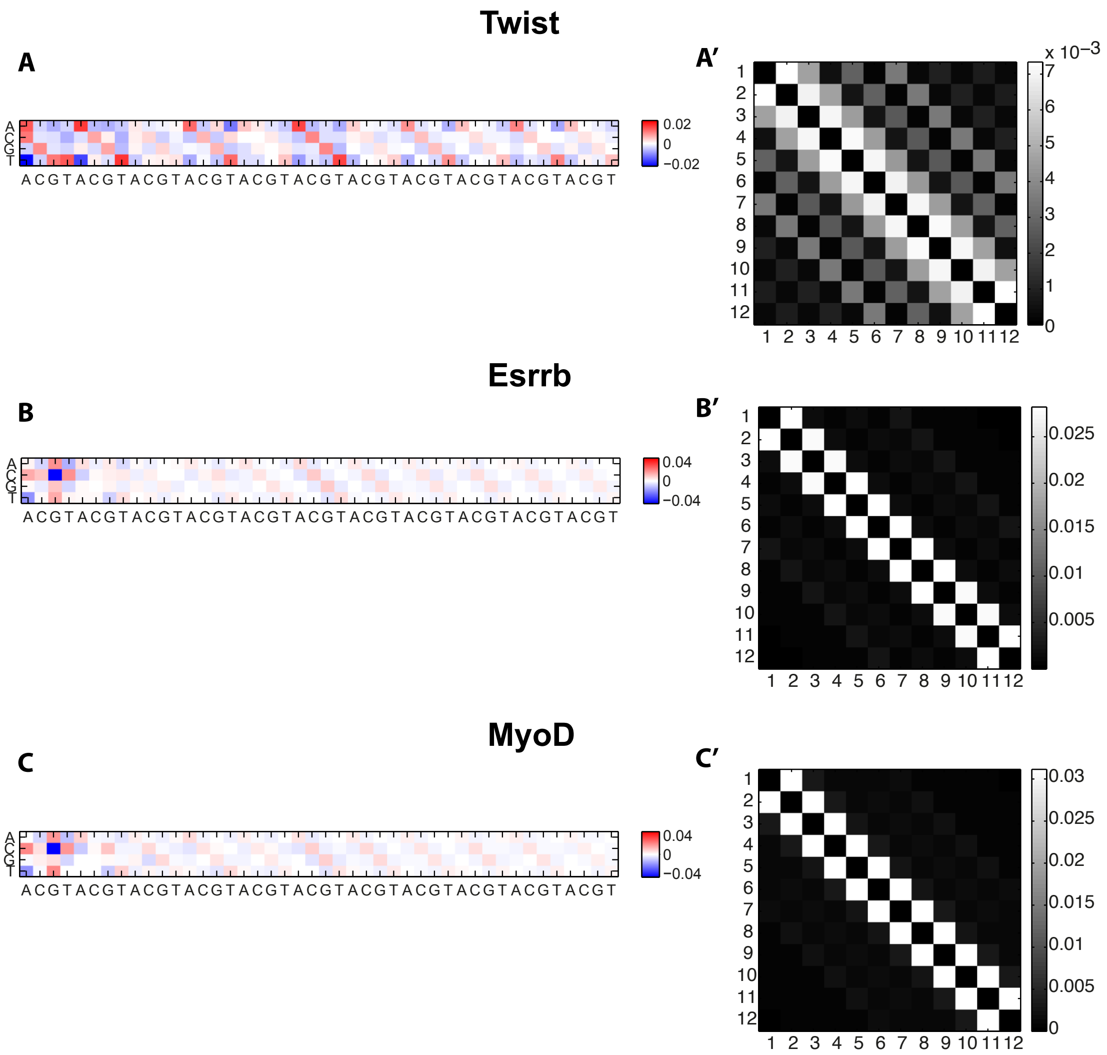

Figure S5. Background correlations. (A,B,C) Heat maps showing the correlations between nucleotides in the ChIP data of the 3 factors from the main text. Because of translation invariance, we only show the correlations between a nucleotide (rows) and the next nearest (first four columns) to farthest (last four columns) nucleotides, using the binding site length of $L = 12$. We see in the Drosophila data the appreciable presence of repeated sequences (of type AA, TT, CC, and GG). In the mammalian data sets, we observe the known CpG depletion. (A',B',C') Corresponding heat maps showing the values of the Normalized Direct Information between pairs of nucleotides.

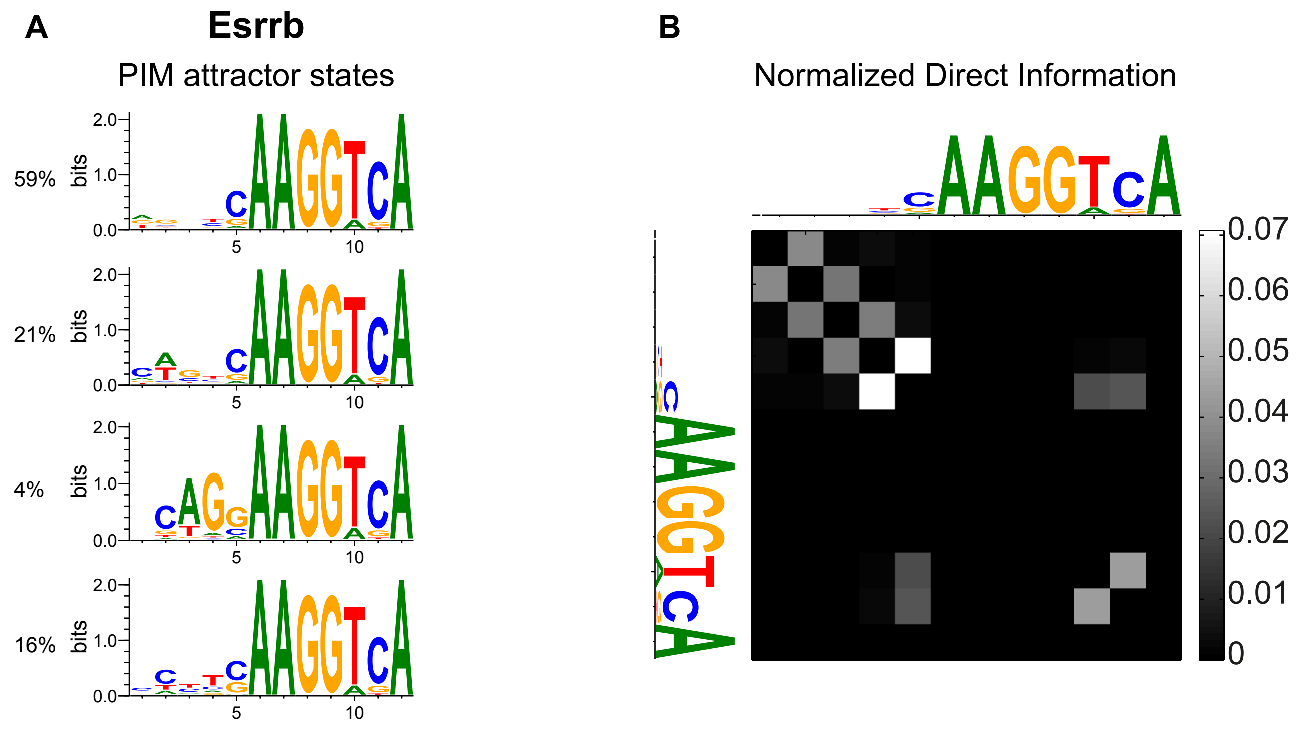

Figure S6. Variable spacer length We learned a PIM for Esrrb including the 4 flanking nucleotides on the left of the main motif. (A) The metastable states of this model show a feature not captured in the main text where binding sites are defined symmetrically around the center of mass of the information content: namely a 'CAG' trinucleotide with variable spacer length from the main motif. This feature is apparent in the first 3 logos shown here. (B) The contribution of this trinucleotidic interaction to the Direct Information is captured through strong direct links between the 4 flanking nucleotides, showing that the PIM is implicitly able to capture higher order correlations. Logos from the PWM model are surrounding the heatmap for clarity.

Supporting Tables

Table S1. Comparison between initial PWMs and $L = 12$ PWMs. Bottom rows correspond to the 6 factors that are satisfactorily described by the PWM model. Information content is in bits.

Name	Reference	Initial length	Initial info	Final info	Loss of info
Bin	Ref. [32] of the main text	12	12.3038	12.3038	0
Mef2	Ref. [32] of the main text	11	9.802	9.802	0
Twi	Ref. [32] of the main text	12	10.766	10.766	0
E2f1	`JASPAR_MA0024.1_E2F1`	8	10.6909	10.6909	0
Esrrb	`JASPAR_MA0141.1_Esrrb`	12	14.2211	14.2211	0
Klf4	`JASPAR_MA0039.2_Klf4`	10	11.358	11.358	0
Nanog	`TRANSFAC_V$NANOG_01`	12	13.1735	13.1735	0
N-Myc	`TRANSFAC_V$NMYC_01`	12	10.4726	10.4726	0
Oct4	`TRANSFAC_V$OCT4_01`	15	17.9438	15.649	−2.29476
Sox2	`TRANSFAC_V$SOX2_Q6`	16	11.2259	10.7835	−0.442423
Tcfcp2l1	`JASPAR_MA0145.1_Tcfcp2l1`	14	11.9982	9.00071	−2.99754
Zfx	`TRANSFAC_V$ZFX_01`	16	9.71554	9.18774	−0.527802
C/EBP-beta	`TRANSFAC_V$CEBPB_02`	14	8.47114	8.35577	−0.115366
CTCF	`TRANSFAC_V$CTCF_01`	20	15.5422	13.4622	−2.08002
E2f4	`TRANSFAC_V$E2F4DP2_01`	8	11.036	11.036	0
Fosl1	`JASPAR_MA0099.1_Fos`	8	10.2943	10.2943	0
Max	`JASPAR_MA0058.1_MAX`	10	11.3238	11.3238	0
MyoD	`TRANSFAC_V$MYOD_Q6`	10	9.25668	9.25668	0
Myog	`TRANSFAC_V$MYOGENIN_Q6`	8	9.50246	9.50246	0
NRSF	`TRANSFAC_V$NRSF_01`	21	23.3918	15.0718	−8.31997
TCF3	`TRANSFAC_V$TCF3_01`	12	12.9923	12.9923	0
USF1	`JASPAR_MA0093.1_USF1`	7	10.5008	10.5008	0
c-Myc	`JASPAR_MA0147.1_Myc`	10	10.5487	10.5487	0
SRF	`JASPAR_MA0083.1_SRF`	12	18.1745	18.1745	0
STAT3	`JASPAR_MA0144.1_Stat3`	10	14.5568	14.5568	0
Bap	Ref. [32] of the main text	12	10.3746	10.3746	0
Tin	Ref. [32] of the main text	10	10.623	10.623	0
Smad1	`TRANSFAC_V$SMAD1_01`	12	11.0164	11.0164	0
